# Quantitative amino acid analysis by liquid chromatography‐tandem mass spectrometry using low cost derivatization and an automated liquid handler

**DOI:** 10.1002/jmd2.12080

**Published:** 2019-11-12

**Authors:** William S. Phipps, Eric Crossley, Richard Boriack, Patricia M. Jones, Khushbu Patel

**Affiliations:** ^1^ Department of Pathology University of Texas at Southwestern Medical Center Dallas Texas; ^2^ Chemistry, Metabolic Disease and Mass Spectrometry Laboratories Children's Health Dallas Texas

**Keywords:** amino acid, inborn errors of metabolism, isotope‐coded derivatization, liquid chromatography, mass spectrometry, triple quadrupole

## Abstract

Amino acid analysis is central to newborn screening and the investigation of inborn errors of metabolism. Ion‐exchange chromatography with ninhydrin derivatization remains the reference method for quantitative amino acid analysis but offers slow chromatography and is susceptible to interference from other co‐eluting compounds. Liquid‐chromatography tandem mass spectrometry (LC‐MS/MS) provides a rapid and highly specific alternative, but sample preparation is frequently laborious and sometimes cost prohibitive. To address these limitations, we validated an LC‐MS/MS method using the aTRAQ Reagents Application Kit with a modified protocol consuming only half reagents. Adequate performance for clinical specimen measurement of 26 amino acids with high clinical relevance was achieved. An automated liquid handler and modified calibration and normalization approaches were used to ensure reproducible assay performance. Linear measurement between 5 and 2000 μM was achieved for most analytes despite use of a small, 10 μl sample size. Overall the method achieved near substantially improved throughput and enabled use of smaller samples volumes for batched analyses of clinical samples.

**SYNOPSIS**

Amino acid analysis using aTRAQ reagents is feasible at half reagent volumes using an automated liquid handler for precision handling of small samples.

## 1 INTRODUCTION

Quantitative amino acid analysis is frequently required for the diagnosis and monitoring of inherited metabolic disorders. Conventional ion‐exchange chromatography (IEX) offers slow analyte separation (2‐3 hours per sample), hindering throughput, particularly when calibrators and quality control samples must also be analyzed. Meanwhile, faster, reverse‐phase liquid chromatography (RPLC) methods may require substantial optimization and full amino acid separation is not guaranteed.^1^, ^2^, ^3^ Due to the reliance on UV‐based detection, both RPLC and IEX platforms are subject to optical inferences.^4^, ^5^ Applying mass spectrometry (MS)‐based detection to amino acid analysis largely overcomes challenges related to speed while enhancing specificity of analysis.^6^

Numerous MS methods have been implemented using different types of separation and mass analyzers, with and without derivatization.^7^, ^8^, ^9^, ^10^, ^11^, ^12^ Between MS methods, key differentiators affecting cost‐per‐test are derivatization and calibration reagents. Low cost amine derivatization using butylation has longstanding use in amino acid analysis, but performance is variable.^13^, ^14^, ^15^ The application of light and heavy isotope‐coded derivatization (ICD) reagents is one alternative that is gaining more wide‐spread adoption in clinical laboratories.^12^, ^16^, ^17^, ^18^ A key advantage is broad internal standard coverage. Amino acids in the patient samples are derivatized with a mass tag, and combined at the end of sample processing with an internal standard amino acid mix pre‐derivatized with a mass tag of identical structure but alternative isotopic makeup. The use of light and heavy tags enables discrimination by MS/MS. ICD kits are commercially available, including the aTRAQ format (SCIEX), with a mass difference of 8 amu between derivatized amino acid targets and quantifying internal standards. Pre‐synthesized proprietary derivatization reagents may be prohibitively expensive compared to traditional methods when used per manufacturer's recommendations. Here, we present a method for quantitative analysis of 26 clinically relevant amino acids using derivatization via aTRAQ reagents, using a modified protocol to achieve near cost equivalence with our current IEX and ultra‐performance liquid chromatography (UPLC) methods. In the modified procedure we report here, all reagent volumes are halved per sample. Additionally, to ensure more consistent quantitation and better account for sample extraction variability between amino acids in different plasma samples, we incorporated a mix of 17 isotopically labeled amino acids added at the beginning of sample preparation for normalization. A liquid handler semi‐automates the extraction and derivatization process cutting down on the manual sample preparation time and ensuring consistency of handling between samples.

## 2 MATERIALS AND METHODS

### 2.1 Reagents

Amino acid analytical standards and lyophilized S‐(2‐Aminoethyl)‐l‐cysteine hydrochloride (SAEC), glutamine, l‐asparagine, argininosuccinic acid, O‐phosphorylethanolamine, l *‐allo*‐isoleucine, l‐homocitrulline, and l‐norvaline were purchased from Sigma and Pickering. A stable isotope (^13^C,^15^N) labeled mixture of 17 amino acids (MSK‐A2) was purchased from Cambridge Isotope Laboratories. The aTRAQ Kit for Amino Acid Analysis was purchased from SCIEX. Seraprep and Uriprep deproteinization reagents were obtained from Pickering Laboratories. Ninhydrin derivatization reagent (80‐2117‐64) and other IEX associated materials were purchased from Biochrom Ltd. The MassTrak AAA Derivatization Kit containing 6‐aminoquinolyl‐N‐hydroxysuccinimidyl carbamate (AQC, 186004095) reagent and other UPLC‐associated materials were purchased from Waters.

### 2.2 Sample preparation for liquid chromatography‐mass spectrometry

Amino acid amine derivatization was performed using aTRAQ reagents, with partial automation via a repurposed QIAGEN QIAgility instrument (configuration detailed in Figure S1). Sample preparation was conducted according to kit manufacturer's recommended protocol with modifications. 10 μl of 250 μM (commercial 2500 μM stock diluted 10× in 0.1 M HCl) isotopically labeled 17 amino acid mixture was added to each 10 μl test sample. For deproteinization, 5 μl of 10% sulfosalicylic acid (SSA) was added to the sample‐internal standard mix. After vortexing and centrifugation, 5 μl of deproteinized sample was diluted in 20 μl of the supplied aTRAQ Labeling Buffer. For derivatization, 5 μl of the sample and labeling buffer mixture was then combined with 2.5 μl of aTRAQ reagent, the latter diluted first in 70 μl isopropanol per manufacturer's instructions. Following a 40 minutes incubation at room temperature, 2.5 μl hydroxylamine (kit reagent) was added to mixture for reaction quenching for 15 minutes at room temperature. To enable *allo*‐Ile quantification, an additional 5 μl of the underivatized sample diluted in labeling buffer was added to the mixture. The final sample was diluted with 180 μl water.

### 2.3 Liquid chromatography‐mass spectrometry

LC‐MS/MS analysis was performed on an Agilent 1290 UPLC system, SCIEX 4500 tandem mass spectrometer, and ESI source operated in positive ionization mode. Chromatography was performed using a specialized C18 column (SCIEX 4374841, dimensions 5 μm, 4.6 mm × 150 mm) at temperature of 50°C with 0.1% formic acid and 0.01% heptafluorobutyric acid (HFBA) in water (mobile phase A) and 0.1% formic acid and 0.01% HFBA in methanol (mobile phase B), at a rate of 1.0 ml/min using the gradient in Table S1. Sample injection volume was 20 μl. The mass spectrometer was operated in selective reaction monitoring (SRM) mode using the following settings: ion spray voltage 3500 V, entrance potential 10 V, declustering potential 35 V, collision cell exit potential 10 V. The Q1/Q3 transitions (Table S2) for target analytes were provided by the kit manufacturer based on expected fragmentation within the derivatization tag. The Q1 masses for internal standards (stable‐isotope labeled structural analogs) were calculated based on the ^13^C,^15^N labeling in native amino acid structure. An additional transition for underivatized stable‐isotope (^13^C,^15^N) labeled isoleucine was determined by optimization during direct infusion. Instrument control, data acquisition, and processing were performed using Analyst (SCIEX) software, 1.6.3.

### 2.4 Quantification of amino acids

The LC‐MS/MS method utilized a single point, 250 μM external calibration sample containing all target amino acids analyzed with each run. Results for individual amino acids were normalized to 1 of 17 stable‐isotope (^13^C,^15^N labeled) amino acid analogs added at the beginning of sample preparation (Figure S2). For amino acids missing an isotopically labeled structural analog (ASA, Asn, Cit, Gln, homocysteine, Orn, Tau, Trp, *allo*‐Ile), an internal standard was selected based on proximity in retention time and performance in empirical testing (Table 1). Signals for underivatized Ile and *allo*‐Ile were normalized against underivatized ^13^C,^15^N labeled Ile. The aTRAQ kit internal standard (SCIEX 4442688) was not utilized.

**Table 1:** Linearity and precision data

		Standard curve^c^					Intrarun precision (%CV)^d^			Inter‐run precision (%CV)		
AA^a^	IS^b^	LLOQ	%CV at LLOQ	ULOQ	%CV at ULOQ	*R* ^2^	Low	High	Plasma	Low	High	Plasma^e^
Ala	Ala	5	16.0	2000	3.1	1.00	1.8	0.88	3.1	6.6	3.6	5.8
Arg	Arg	5	14.6	2000	1.1	1.00	5.1	7.1	4.5	6.3	4.9	8.4
Asa	Thr	10	15.7	2000	1.2	1.00	1.3	4.4	6.8	5.2	4.6	NP
Asn	Thr	5	7.0	2000	2.2	1.00	3.4	2.5	4.7	2.9	2.4	13
Asp	Asp	5	2.4	2000	1.7	1.00	1.5	4.4	4.2	9.8	4.8	16.2
Cit	Gly	5	2.8	2000	2.2	1.00	2.2	7.6	4.5	9.2	4.5	3.7
Cys	Cys	2.5	5.1	1000	6.9	0.99	2.6	2.1	2.9	4.3	4.1	8.5
Glu	Glu	5	7.8	2000	5.3	1.00	4.9	3.9	3.1	3.3	3.9	7.7
Gln	Gly	5	13.0	2000	2.6	1.00	1.9	1.4	10.4	7.1	5.7	11.2
Gly	Gly	5	12.0	2000	2.3	0.93	2.8	3.1	5.1	6.2	8.5	5
Hct	Lys	5	5.9	2000	5.8	1.00	NP	NP	NP	4.8	7.1	4.7
Hcy	Pro	5	19.1	2000	1.1	1.00	2.7	3.1	NP	7.5	7.1	NP
His	His	5	8.8	2000	0.8	1.00	4.2	3.1	3.9	8.9	3.2	2.6
Ile	Ile	5	5.3	2000	11.5	0.99	0.8	1.4	4.9	5.4	5.7	6.5
Leu	Leu	10	2.5	2000	8.4	0.99	9.3	4.2	5.3	8.5	5.6	5.7
Lys	Lys	5	5.3	2000	2.9	1.00	2.3	2.2	5.5	5.4	4.3	2.7
Met	Met	5	1.3	2000	2.7	1.00	7.1	1.9	2.7	7.4	4.5	4.1
Orn	Asp	5	5.5	2000	6.1	0.99	1.9	13.6	5.6	7.5	7.9	5.2
Phe	Phe	5	3.4	2000	11.7	0.99	1.3	2.7	4.8	6.2	4.7	2.8
Pro	Pro	5	6.7	2000	1.4	1.00	6.7	2.4	3	8.5	4.1	3.0
Ser	Ser	5	9.0	2000	5.0	1.00	0.81	4.6	5.2	5.9	3.5	8.1
Tau	Gly	5	6.0	2000	0.5	0.99	1.7	3.1	10.4	6.1	6.9	7.4
Thr	Thr	10	4.2	2000	2.1	1.00	7.2	1.2	2.3	6.1	3.8	2.4
Trp	Gly	5	0.7	2000	8.5	0.99	4.1	2.7	5.4	8.9	3.6	5.2
Tyr	Tyr	5	9.5	2000	5.8	1.00	4.3	2.5	0.9	5.5	5.5	1.3
Val	Val	5	8.7	2000	4.0	1.00	0.49	1.2	3.1	5.5	3.5	1.1
u‐Ile	u‐Ile	25	8.0	2000	6.2	0.99	2.9	1.4	NP	6.8	2.1	NP
u‐*allo*	u‐Ile	5	10.2	500	1.9	1.00	3.2	4.1	NP	10.8	0.7	NP

a Cys was measured as cystine. Asa, argininosuccinic acid; Hct, homocitrulline; Hcy, homocystine; u‐Ile, underivatized isoleucine; u‐*allo*, underivatized *allo*‐isoleucine. The referenced 26 clinically relevant targets in the manuscript include *allo*‐isoleucine, isoleucine (once, as derivatized or underivatized), and excludes taurine.

b ^13^C,^15^N‐labeled (Cambridge Isotope mix MSK‐A2).

c Standard curves utilized Sigma standards (A6407, A6282, and lyophilized standards) prepared in 0.1 M HCl. The R^2^ values reflect fit to linear curve over entire 5 to 2000 μM tested range (2.5‐1000 μM for Cys, 5‐500 μM for *allo*‐Ile).

d All %CV calculations used standard deviation estimations based on triplicate sample analysis. Generally, target concentrations were 50 μM for “low” QC and 250 μM for “high” QC.

e NP, not performed.

### 2.5 LC‐MS/MS method validation

Analytical measurement range (AMR) was determined via analysis of amino acid standard mixes prepared from 5 to 2000 μM (2.5‐1000 μM for cystine) in 0.1 M HCl. The standard curves and a blank sample (0.1 M HCl) were prepared and analyzed in triplicate, with blank analyzed by LC‐MS/MS after the 2000 μM standard to assess carryover. Separate standard curves were prepared for underivatized Ile and *allo*‐Ile analysis, to assess for capacity to measure low levels *allo*‐Ile (10 μM) in the presence of increasing Ile (5, 25, 500, 1000, and 2000 μM) and linearity of *allo‐*Ile (5, 10, 50, 100, and 500 μM) in the presence of fixed, high Ile (500 μM). Inter and intrarun precision (%CV) were determined for “low” and “high” concentration control samples (in 0.1 M HCl, see Section 3), patient plasma (a sample from the group of 30 below), and pooled matrix samples (latter experiment described in Supporting Information). Correlations with conventional IEX and UPLC platforms for 20 amino acid targets were examined using leftover heparinized blood plasma patient samples (n = 30) sent to the clinical laboratory for amino acid analysis. Specimens were either analyzed fresh or stored at −20°C for <72 hours prior to analysis. Amino acids excluded from this analysis were either rare amino acids (*allo*‐Ile, argininosuccinic acid, homocitrulline, and homocystine), or targets with concentrations highly dependent on preanalytical factors (ie, Glu, Cys, and Asp) since storage time prior to analysis could not equalized between methods due to workflow of the laboratory. Of note, Gln and Asn are subject to pre‐analytical conversion to Glu and Asp but occur at naturally higher endogenous concentrations such that small conversions do not necessarily limit correlation. Matrix effects and ion suppression (%) were estimated in each patient sample based on comparison of the internal standard signals spread across the chromatogram with those in the external standard (in 0.1 M HCl). Accuracy in spiked plasma was assessed for all amino acids, using plasma negative for homocystine and argininosuccinic acid by IEX. Accuracy in spiked urine was assessed for Arg, Lys, and Orn, and spiked CSF for Gly and Ser. Interference studies consisted of lipemia mixing studies in which six plasma samples were analyzed for triglycerides and the 5 highest samples were mixed in 1:1 ratio with the sample with lowest measured triglyceride level. The individual samples and mixed samples were then analyzed by LC‐MS/MS.

### 2.6 Specimen analysis by liquid chromatography

Analysis of patient specimens by liquid chromatography was performed using either IEX with post‐column ninhydrin derivatization on a Biochrom 30+ Amino Acid Analyzer (n = 19) or UPLC on a MassTrak Amino Acid Analysis system (n = 11). Sample preparation for IEX consisted of: (a) mixing specimens (eg, 100 μl plasma) in 1:1 ratio with 250 μM SAEC internal standard in a SSA‐based deproteinization reagent (Seraprep or Uraprep), (b) vortex mixing and centrifugation (2 minutes, 16 000*g*) and (c) transfer of supernatant for analysis. Sample preparation for UPLC consisted of: (a) sample (50 μl) deproteinization in SSA‐based deproteinization reagent, (c) vortex mixing/centrifugation, (c) dilution of supernatant in borate buffer containing norvaline internal standard, and (d) heated derivatization using the AQC reagent per manufacturer's instructions and transfer of mixture for analysis.

### 2.7 Data analysis

Amino acids analyzed by LC‐MS/MS were quantified using MultiQuant 3.0.2. (SCIEX). Statistical analyses were performed in Microsoft Excel and GraphPad Prism software. For standard curves prepared from 5 to 2000 μM, simple linear regression was used to compare LC‐MS/MS result and expected concentration. The lower limits of quantitation (LLOQ) and upper limits of quantitation (ULOQ) were determined as the lowest and highest concentrations for which at least 2 of 3 results were within ±20% of target, %CV was <20%, and chromatography was of sufficient quality for reliable quantitation. Carryover (%) was determined as: (blank peak area)/(2000 μM standard area) × 100%. For correlation with conventional IEX and UPLC platforms, Deming regression was used to compare LC‐MS/MS and conventional method results. For spiked matrix measurements, a baseline non‐spiked concentration was determined by LC‐MS/MS, and expected spiked concentrations extrapolated from the baseline value. Ion suppression (%) was estimated in each patient sample using the isotopically labeled compounds across the chromatogram as: (I.S. area in external standard − I.S. area in plasma sample)/(I.S. area in external standard in 0.1 M HCl) × 100%.

## 3 RESULTS

### 3.1 Linearity, analytical measurement range, carryover, and precision

Linear regression of the LC‐MS/MS method on target concentration demonstrated coefficient of determination (*R* ^2^) ≥ 0.99 for all derivatized amino acid targets over the 5 to 2000 μM tested range (2.5‐1000 μM for Cys; Table 1), as well as in the separate analysis for both underivatized Ile and *allo*‐Ile over the tested ranges (5‐2000 μM for Ile and 5‐500 μM for *allo*‐Ile). LLOQ was 5 μM (2.5 μM for Cys) for all derivatized amino acids, except for argininosuccinic acid (10 μM), leucine (10 μM), and threonine (10 μM). ULOQ was 2000 μM for all derivatized targets (1000 μM for cystine). LLOQ was 25 μM for underivatized Ile (10 μM not tested) and 5 μM for *allo*‐Ile. ULOQ were 2000 and 500 μM (highest concentrations tested), respectively. Complete standard curve results appear in Tables S3 and S4. Carryover was not detected for most analytes or was significantly <1% (data not shown). Coefficients of variation (%CV) for derivatized amino acids were less than 10% (frequently <5%), with the following exceptions: inter‐run plasma Asn (13.0%), Asp (16.2%), and Gly (11.2%), and intrarun plasma Gly (10.4%). Analysis of high and low QC samples containing underivatized *allo*‐Ile and Ile demonstrated %CV <15%. Precision data appear in Tables S5, S6, and for pooled matrix study later in Table S13.

### 3.2 Comparison of LC‐MS/MS method with conventional IEX and UPLC methods

Method correlation data is summarized in Table 2 (complete results in Table S7) including Pearson's *R* values based on linear correlation of LC‐MS/MS with conventional testing. Each clinical specimen was tested in the laboratory using either IEX or UPLC. Strong correlation (*R* > 0.95) was determined for 12 of 20 targets (Ala, Cit, Ile, Leu, Lys, Met, Phe, Tau, Thr, Tyr, and Val) when comparing against combined IEX and UPLC tested clinical specimens (n = 30, Figure S3A). For Arg, Gly, and Orn *R* > 0.95 was observed only when restricting analysis to IEX specimens (n = 19, Figure S3B), and for Asn, Gln, His, Ser, and Trp when restricting analysis to UPLC specimens (n = 11, Figure S3C). Estimation of combined matrix effects and ion suppression in these experiments appears in Table S8. Average signal suppression ranged from 23.8% to 35.9%.

**Table 2:** Method correlation and accuracy (plasma)

	Correlation with clinical specimen testing^a^									
	Combined IEX and UPLC specimens (n = 30)		IEX specimens only (n = 20)		UPLC specimens only (n = 10)		Accuracy (spiked plasma)^b^			
AA	Range (μM)	*R*	Range (μM)	*R*	Range (μM)	*R*	n	Range (μM)	Within ±20%	Within ±10%
Ala	107‐508	**0.98**	140‐447	**0.99**	107‐508	**0.99**	6	265‐697	100%	100%
Arg^c^	34‐206	0.93	51‐152	**0.98**	34‐206	0.90	5	128‐1313	100%	100%
ASA	NA	NA	NA	NA	NA	NA	6	25‐500	100%	50%
Asn	13‐94	0.94	22‐94	0.91	13‐67	**1.00**	6	44‐419	67%	67%
Asp	NA	NA	NA	NA	NA	NA	6	11.2‐386.2	83%	67%
Cit	4‐47	**0.98**	10‐47	**0.98**	4‐21	**0.97**	6	13.2‐388.2	100%	33%
Cys	NA	NA	NA	NA	NA	NA	6	17.8‐205.3	83%	67%
Gln	246‐891	0.94	364‐891	0.92	246‐793	**0.97**	6	561‐1231.3	100%	100%
Glu	NA	NA	NA	NA	NA	NA	6	36‐433	100%	83%
Gly^c^	152‐587	0.94	184‐587	**0.99**	152‐427	0.91	6	219‐594	100%	100%
HCY	NA	NA	NA	NA	NA	NA	5	5‐1250	100%	80%
His	42‐118	0.92	42‐97	0.80	46‐118	**0.99**	5	105‐1301	100%	80%
HCT	NA	NA	NA	NA	NA	NA	8	6‐2000	100%	75%
Ile	29‐175	**0.99**	21‐174	**0.99**	29‐135	**0.99**	6	43‐418	100%	100%
Leu	44‐863	**1.00**	44‐863	**1.00**	94‐299	**0.99**	6	76‐608	100%	83%
Lys^c^	64‐251	**0.96**	64‐251	**0.99**	67‐230	**0.96**	5	215‐1357	100%	100%
Met	8‐73	**0.98**	9‐46	0.94	8‐73	**0.99**	6	18‐393	100%	83%
Orn^c^	43‐206	0.89	43‐110	**0.95**	56‐206	0.86	5	157‐1328	100%	100%
Phe	38‐404	**0.99**	38‐404	**1.00**	73‐202	**0.98**	6	36‐411	100%	83%
Pro	64‐737	**0.99**	77‐737	**0.99**	64‐312	**0.98**	6	209‐584	100%	83%
Ser^c^	84‐268	0.94	118‐268	**0.96**	84‐237	**0.98**	6	153‐528	100%	83%
Tau	22‐177	**1.00**	24‐152	**0.99**	22‐70	**0.98**	6	51‐502	100%	67%
Thr	40‐374	**0.99**	57‐374	**0.99**	40‐231	**0.98**	6	114‐489	100%	100%
Trp	30‐88	0.93	30‐88	0.87	34‐83	**0.98**	5	59‐1277	100%	60%
Tyr	21‐286	**1.00**	21‐286	**1.00**	29‐96	**0.99**	6	46‐421	100%	83%
Val	84‐931	**0.99**	84‐931	**1.00**	151‐485	**0.96**	6	143‐666	100%	100%

a Clinical specimens were analyzed by either IEX or UPLC. Correlations with Pearson *R* >0.95 in bold.

b The “Within ±20%” and “Within ±10%” metrics reflect number of results (out of 5‐8 total) within a certain percentage of error from calculated expected concentration for the analyte. Individual results appear in Table S9. Underivatized Ile and *allo*‐Ile in plasma were subject to separate accuracy analysis (detailed in Table S12).

c Accuracy in spiked specimens was also tested in urine or CSF (Tables S10 and S11) for Arg, Gly, Lys, Orn, and Ser.

### 3.3 Accuracy of derivatized amino acids in spiked plasma, urine, and CSF

Using spiked plasma, 148 of 152 (97%) LC‐MS/MS results were within ±20% of expected concentration at various levels of analytes, with a majority of results within ±10% (summarized in Table 2, complete results Table S9). Using spiked urine, LC‐MS/MS results for Arg, Lys and Orn results were within ±20% of expected concentration (Table S10) and all CSF glycine and serine results were within ±20% of expected with 1 exception (recovery equaled 122% of expected for a Serine CSF result) for samples within the defined AMRs (Table S11).

### 3.4 Isoleucine vs *allo*‐Isoleucine in plasma

Complete baseline chromatography separation of underivatized *allo*‐Ile and Ile was not achieved using the described gradient (Figure S4) or the longer manufacturer recommended protocol (not shown). Separation was adequate, however, to discriminate *allo*‐Ile positive (≥5 μM) and negative samples (Figure S4) and perform separate quantitation. Accuracy in plasma for underivatized Ile and *allo*‐Ile was confirmed via comparison with IEX for five patient specimens positive for *allo*‐Ile (at 7.1, 7.4, 9.2, 16.2, and 400.8 μM), and analysis of four additional spiked plasma samples (Table S12).

### 3.5 Miscellaneous studies

Results for an additional precision and matrix suppression experiment utilizing pooled plasma, urine and CSF appears in Table S13 and lipemia mixing studies for plasma in Table S14. Imprecision (%CV) across 15 replicates was less than 10% for most analytes across the three matrices. Higher imprecision was observed for the underivatized leucine derivatives (18.4% for *allo*‐Ile 16.7%) in plasma, Cys in plasma (11.3%), as well as ornithine in urine (12.1%).

Decreases in analyte signal based on intensity of internal standard analytes were generally greater in plasma samples compared to urine and CSF. Significant effects on results were not observed in lipemia mixing studies.

## 4 DISCUSSION

Use of the aTRAQ reagents has been previously described as an accessible approach to LC‐MS/MS‐based quantitative amino acid analysis.^11^, ^17^, ^18^ However, in contrast to a previous report,^18^ use of half reagents was required in our case for per test cost equivalence with current methods. Specific cost benefits of using the described half‐reagent approach will vary from laboratory‐to‐laboratory and will depend on contracts with vendors and pre‐existing availability of liquid handling instruments and technical staff, among other factors. This modification did not significantly reduce performance when applied in combination with a precision liquid handler and a broader internal standard mixture for sample preparation. The aTRAQ method normally relies only on addition of non‐proteinogenic amino acids norleucine (spiked in SSA) and norvaline (spiked in Labeling Buffer) as internal controls before deproteinization and derivatization, respectively. However, in our experience, correction based only these two amino acids did not sufficiently adjust for variable matrix effects between samples at the lower operational volumes. Meanwhile, adding the commercially available ^13^C,^15^N‐labeled amino acid mix did not significantly increase cost since only 1 μl of the stock 2500 μM solution is required per sample.

In addition to halving the reagents and the altered internal standard approach, major sample preparation modifications to the aTRAQ protocol included sample volume reduction from 40 to 10 μl and elimination of a drying step. The reduction in sample volume from 40 to 10 μl included a reduction from 40 to 20 μl based on halving the reagents, followed by further reduction from 20 to 10 μl to make room for internal standard addition, as well as our observations in preliminary studies that very high concentration samples could be running out of derivatization reagent (Figure S5). The drying step is normally employed to evaporate isopropanol from the prepared specimens (and potentially other interferents) prior to LC‐MS/MS analysis. Our procedure uses only 2.5 μl of isopropanol containing derivatization reagent (compared to 5 μl per manufacturer's instructions). Following completion of the derivatization, the samples are left open to the ambient air (approximately 35 minutes) providing adequate exposure for isopropanol evaporation as well as further dilution through the addition of 180 μl water. By bypassing this drying step we may be avoiding previously reported methionine oxidation.^18^

All analytes demonstrated strong correlation (Pearson's *R* ≥ 0.95) with at least one of either IEX or UPLC. Thus, weaker correlations for select amino acids (Arg, Asn, Gln, Gly, His, Orn, Ser, and Trp) were attributable in all cases to lesser agreement with one of the either two reference methods. For example, His (combined *R* = 0.92), suffered weaker correlation with the IEX platform (*R* = 0.80) compared to the UPLC analysis (*R* = 0.99). In contrast, ornithine (combined *R* = 0.89), suffered weaker correlation with the UPLC platform (*R* = 0.86) compared to IEX analysis (*R* = 0.95). The observed discrepancies can be explained by incomplete analyte separation in the optical‐based method used for comparison, as well as relatively narrow spread of concentrations across the clinical samples.

Here, use of repurposed QIAGEN QIAgility liquid handling setup significantly reduced the burden of sample preparation. The granularity in procedural control for a robot designed to do PCR is limited. Nonetheless the handler was able to perform the required small volume manipulations with high precision. Sample preparation can take up to 2 hours, relatively independent of the number of samples. Meanwhile the IEX method requires only minutes of sample preparation per sample but quickly dwarfs the LC‐MS/MS in overall turnaround time due to several hours of chromatography time and requirements related to washing and equilibration (Table 3). In our laboratory, the IEX platform is calibrated upon each startup. Samples are added in queue to be run in sequence with controls at set intervals. As a result, the LC‐MS/MS and IEX platforms offer different, and somewhat complementary workflows.

**Table 3:** Method comparison

Method	Analysis characteristics^a^		Time requirements (min)^b^		
	Comprehensive?	Free from optical interference?	Hands‐on prep (per sample)	Chromatography startup	Chromatography gradient
Ion exchange (IEX)	**Yes**	*No*	5	*>200*	*150*
UPLC‐UV	*No*	*No*	1	*90*	*45*
LC‐MS/MS (w/o handler)	**Yes**	**Yes**	10	**30**	**11**
LC‐MS/MS (w/handler)	**Yes**	**Yes**	4^c^	**30**	**11**

a Analyte coverage for chromatography methods using optical detection will vary from laboratory to laboratory and depends on quality of analyte separation.

b The hands‐on prep times shown assume a 12 sample batch analysis for the UPLC and LC‐MS/MS methods. Chromatography startup reflects time for equilibration, column washing, and so forth.

c The minimum overall sample preparation time for LC‐MS/MS using the handler is about 2 h (incl. ~45 min hands on time) regardless of batch size.

## CONFLICT OF INTEREST

W.P., E.C., R.B., P.J., and K.P. declare they have no conflicts of interest.

## AUTHOR CONTRIBUTIONS

E.C. and W.P. performed setup, initial testing, and adaptation of the described method. W.P., E.C., P.J., and K.P. were involved in validation design. W.P. performed validation experiments and analyzed the data. R.B. performed direct infusion experiments to determine mass spectrometry transitions for isotopically labeled *allo*‐Isoleucine and advised on technical aspects of software and instrument control. W.P. and K.P. wrote the manuscript. E.C., R.B., and P.J. contributed to editing of the manuscript.

## Supporting information


**Figure S1.** Liquid handler configuration for semi‐automated amino acid derivatization
**Figure S2.** Derivatization chemistry and modified internal standard approach
**Figure S3.** Method correlation
**Figure S4.** Representative ion chromatograms
**Figure S5.** Relative norvaline signals in standards prepared from 5 to 2000 μM in 0.1 N HCl

**Table S1.** Chromatography gradient
**Table S2.** Selective reaction monitoring
**Table S3.** Standard curve analysis—derivatized amino acids
**Table S4.** Standard curve analysis—underivatized isoleucine and *allo*‐Ile
**Table S5.** Supplementary precision data—derivatized amino acids
**Table S6.** Supplementary precision data—underivatized isoleucine and *allo*‐Ile
**Table S7.** Method correlation—patient plasma
**Table S8.** Ion suppression/matrix effect data analysis
**Table S9.** Spiked plasma analysis
**Table S10.** Spiked urine analysis
**Table S11.** Spiked CSF analysis
**Table S12.** Unlabeled isoleucine and *allo*‐isoleucine in plasma
**Table S13.** Matrix‐specific precision and signal suppression results
**Table S14.** Lipemia mixing studies (plasma)

## Data Availability

Additional mass spectrometry data, including raw data files (.wiff format) containing the embedded experiments are available.

## References

[1] Peake RWA , Law T , Hoover PN , Gaewsky L , Shkreta A , Kellogg MD . Improved separation and analysis of plasma amino acids by modification of the MassTrak™ AAA Solution Ultraperformance® liquid chromatography method. Clin Chim Acta. 2013;423:75‐82. 23624257 10.1016/j.cca.2013.03.036

[2] Narayan SB , Ditewig‐Meyers G , Graham KS , Scott R , Bennett MJ . Measurement of plasma amino acids by Ultraperformance® liquid chromatography. Clin Chem Lab Med. 2011;49:1177‐1185. 21722078 10.1515/CCLM.2011.200

[3] Freeto S , Mason D , Chen J , Scott RH , Narayan SB , Bennett MJ . A rapid ultra performance liquid chromatography tandem mass spectrometric method for measuring amino acids associated with maple syrup urine disease, tyrosinaemia and phenylketonuria. Ann Clin Biochem. 2007;44:474‐481. 17761035 10.1258/000456307781646012

[4] Prabhakara J , Rodan L , Peake RWA . Now you see it, now you don't: unidentified plasma amino acid peak. Clin Chem. 2016;62:781‐782. 27127245 10.1373/clinchem.2015.246181

[5] Peake R . Commentary. Clin Chem. 2018;64:1437. 30266718 10.1373/clinchem.2018.288126

[6] Kaspar H , Dettmer K , Gronwald W , Oefner PJ . Advances in amino acid analysis. Anal Bioanal Chem. 2009;393:445‐452. 18843484 10.1007/s00216-008-2421-1

[7] Langrock T , Czihal P , Hoffmann R . Amino acid analysis by hydrophilic interaction chromatography coupled on‐line to electrospray ionization mass spectrometry. Amino Acids. 2006;30:291‐297. 16622599 10.1007/s00726-005-0300-z

[8] Kaspar H , Dettmer K , Gronwald W , Oefner PJ . Automated GC‐MS analysis of free amino acids in biological fluids. J Chromatogr B Analyt Technol Biomed Life Sci. 2008;870:222‐232. 10.1016/j.jchromb.2008.06.018 18603486

[9] Poinsot V , Ong‐Meang V , Ric A , Gavard P , Perquis L , Couderc F . Recent advances in amino acid analysis by capillary electromigration methods: June 2015‐May 2017. Electrophoresis. 2018;39:190‐208. 28805963 10.1002/elps.201700270

[10] Armstrong M , Jonscher K , Reisdorph NA . Analysis of 25 underivatized amino acids in human plasma using ion‐pairing reversed‐phase liquid chromatography/time‐of‐flight mass spectrometry. Rapid Commun Mass Spectrom. 2007;21:2717‐2726. 17659659 10.1002/rcm.3124

[11] De Biase I , Liu A , Yuzyuk T , Longo N , Pasquali M . Quantitative amino acid analysis by liquid chromatography‐tandem mass spectrometry: implications for the diagnosis of argininosuccinic aciduria. Clin Chim Acta. 2015;442:73‐74. 25598409 10.1016/j.cca.2015.01.008

[12] Song C , Zhang S , Ji Z , Li Y , You J . Accurate determination of amino acids in serum samples by liquid chromatography‐tandem mass spectrometry using a stable isotope labeling strategy. J Chromatogr Sci. 2015;53:1536‐1541. 25940231 10.1093/chromsci/bmv049

[13] Casetta B , Tagliacozzi D , Shushan B , Federici G . Development of a method for rapid quantitation of amino acids by liquid chromatography‐tandem mass spectrometry (LC‐MSMS) in plasma. Clin Chem Lab Med. 2000;38:391‐401. 10952221 10.1515/CCLM.2000.057

[14] Dietzen DJ , Weindel AL , Carayannopoulos MO , et al. Rapid comprehensive amino acid analysis by liquid chromatography/tandem mass spectrometry: comparison to cation exchange with post‐column ninhydrin detection. Rapid Commun Mass Spectrom. 2008;22:3481‐3488. 18853396 10.1002/rcm.3754

[15] Johnson DW . Analysis of amino acids as formamidene butyl esters by electrospray ionization tandem mass spectrometry. Rapid Commun Mass Spectrom. 2001;15:2198‐2205. 11746885 10.1002/rcm.501

[16] Zhang S , You J , Ning S , Song C , Suo Y‐R . Analysis of estrogenic compounds in environmental and biological samples by liquid chromatography‐tandem mass spectrometry with stable isotope‐coded ionization‐enhancing reagent. J Chromatogr A. 2013;1280:84‐91. 23374368 10.1016/j.chroma.2013.01.045

[17] Held PK , White L , Pasquali M . Quantitative urine amino acid analysis using liquid chromatography tandem mass spectrometry and aTRAQ reagents. J Chromatogr B Analyt Technol Biomed Life Sci. 2011;879:2695‐2703. 10.1016/j.jchromb.2011.07.030 21852206

[18] Filee R , Schoos R , Boemer F . Evaluation of physiological amino acids profiling by tandem mass spectrometry. JIMD Rep. 2014;13:119‐128. 24190798 10.1007/8904_2013_265 PMC4110342

