# Supplementary material for: Quantitative amino acid analysis by liquid chromatography‐tandem mass spectrometry using low cost derivatization and an automated liquid handler
Source: JIMD Rep. 2019 Nov 12;51(1):62–9. doi: 10.1002/jmd2.12080 (PMC7012744; doi:10.1002/jmd2.12080)
Supplement: Supplementary file 1 — Figure S1. Liquid handler configuration for semi‐automated amino acid derivatization Figure S2. Derivatization chemistry and modified internal standard approach Figure S3. Method correlation Figure S4. Representative ion chromatograms Figure S5. Relative norvaline signals in standards prepared from 5 to 2000 μM in 0.1 N HCl [file JMD2-51-62-s001.pdf]

Supplementary Figures

Table of Contents

Figure S1. Liquid handler configuration for semi-automated amino acid derivatization.......................... 2

Figure S2. Derivatization chemistry and modified internal standard approach..………………………… 3

Figure S3. Method correlation……….………..…………………………………...……….……… 4

Figure S4. Representative ion chromatograms…………………….…………..…..……………………...5

Figure S5. Relative norvaline signals in standards prepared from 5 to 2000 μM in 0.1 N HCl……….… 6

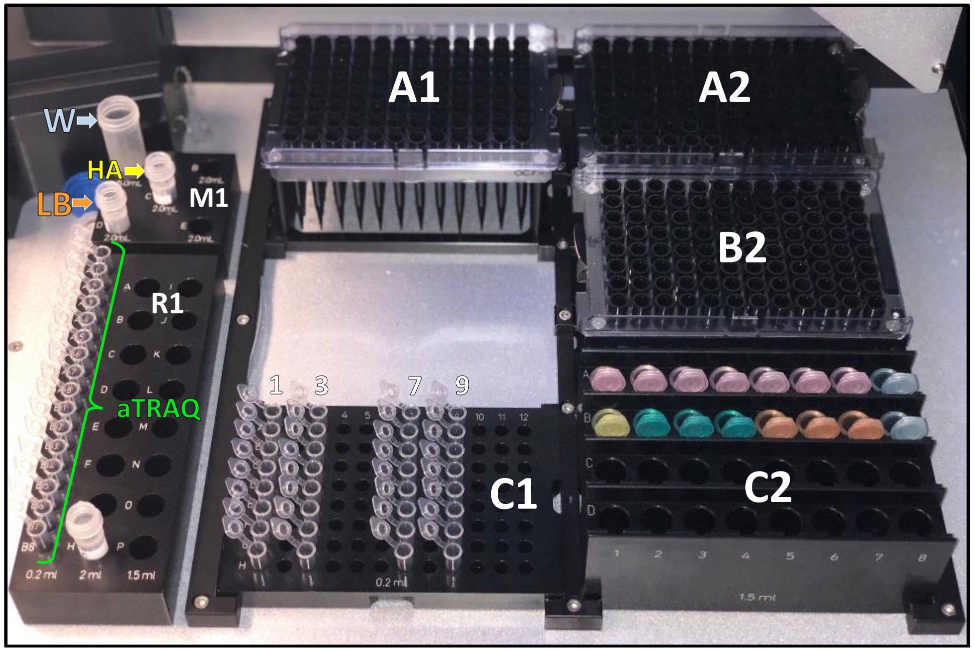

#	QIAgility Instruction	Description of Action
1	Pool samples (2 banks, 25 µl) to 48 well plate (96 well plate, odd columns)	**At Block C1, columns 7 & 9:** Handler combines 20 µl Labeling Buffer (LB) and 5 µl of each deproteinized sample (the latter placed by user at C1, columns 1 & 3).
2	User pause	**At Block R1:** User places tubes, each containing 2.5 µl isopropanol-diluted aTRAQ reagent.
3	Pool samples (1 banks, 5 µl) to Reagent block	**At Block R1:** Handler transfers 5 µl of the buffered samples (from step 1) to the aTRAQ tubes.
4	User pause	User closes the aTRAQ reaction tubes and underivatized buffered sample tubes (leftover at C1, columns 7 & 9).
5	Pause for 00:40:00 hh:mm:ss	Incubation (for derivatization)
6	User pause	User opens the reaction tubes and underivatized buffered sample tubes.
7	Pool samples (1 banks, 2.5 µl) to Reagent block	**At Block R1:** Handler adds 2.5 µl hydroxylamine (HA) to each derivatization reaction.
8	Pause 00:15:00 hh:mm:ss	Incubation (for quenching)
9	Pool samples (1 banks, 5 µl) to Reagent block	**At Block R1:** Handler adds 5 µl of underivatized buffered samples (from C1, columns 7 & 9) to the corresponding quenched derivatization reactions.
10	Pool samples (1 banks, 13 µl) to 32 well Flip Cap plate @ C2	**At Block C2:** Handler transfers 13 µl of the quenched derivatization reactions (from R1) to 1.5 ml Eppendorf tubes.
11	Sample dilution of Final Samples and Water	**At Block C2:** The handler adds 180 µl reagent water (W) to each tube

Figure S1. Liquid handler configuration for semi-automated amino acid derivatization. The user performs sample deproteinization outside the handler (Qiagen QIAgility), and places the deproteinized samples at Block C1 in columns 1 and 3 (left image). Position of materials and reagents for further sample preparation include 200 µL tips at A1, 50 µl tips at A2 and B2, Labeling Buffer (LB), hydroxylamine (HA), and water (W) at M1, empty PCR tube strips at C1 in columns 7 and 9, and 1.5 mL Eppendorf tubes at C2. PCR tubes strips containing aTRAQ reagent are placed at R1 immediately before derivatization to avoid evaporation of isopropanol. The QIAgility instructions (right table) are displayed in the format used in the QIAgility software. The QIAgility liquid handler primarily performs "Sample Pooling" operations. Sample Pooling instructions include entry of a "Target reaction plate" and the "Samples" to be pooled at the target. The associated QIAgility project file is available upon request for interested users. The described configuration enables analysis of up to 12 patient samples, in addition to the external standard, a blank, high control and low control.

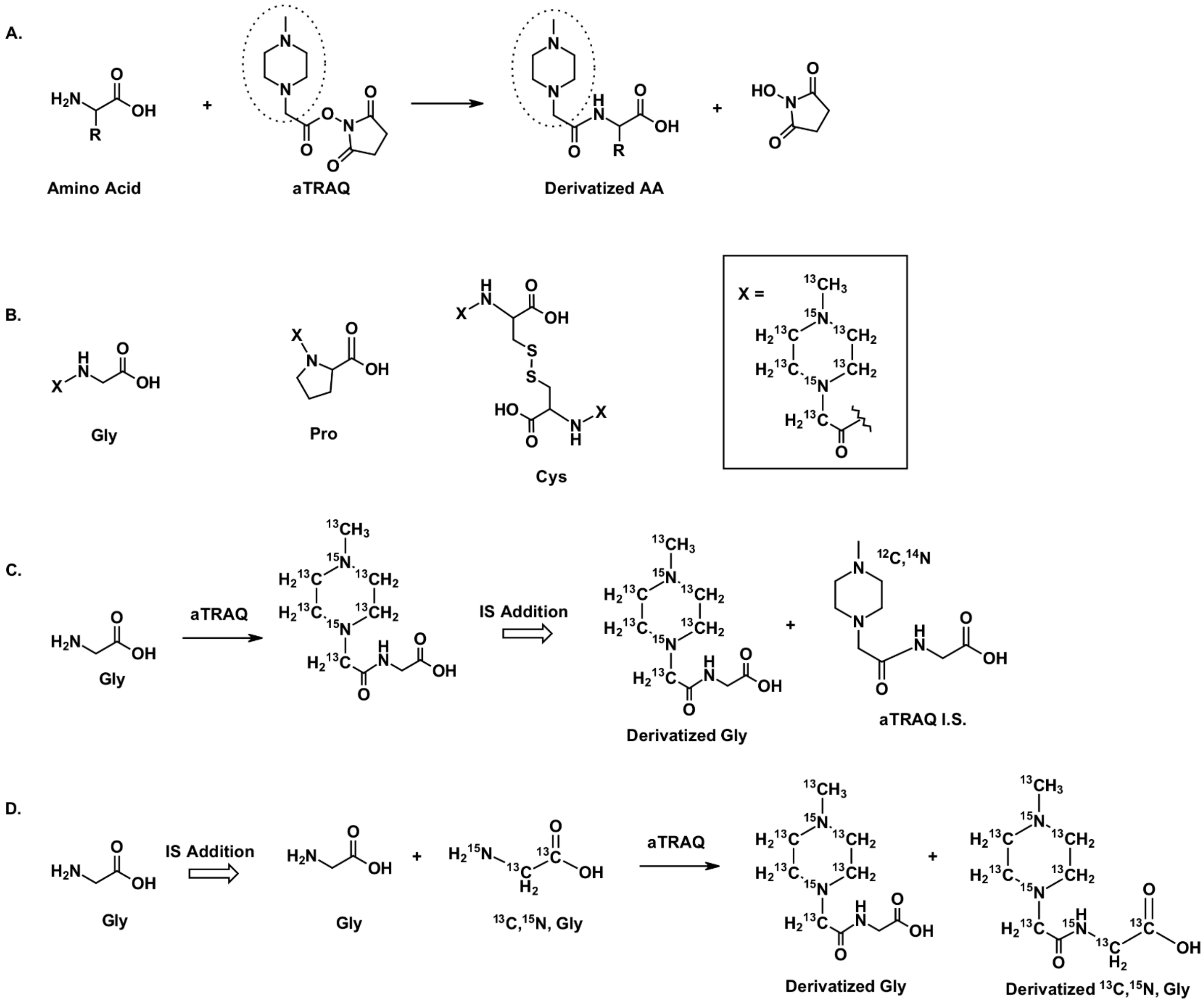

Figures S2. Derivatization chemistry and modified internal standard approach. aTRAQ sample derivatization kit uses stable-isotope coded labeling of the amino group **(A)**. Specimen amino acids are labeled with mass tag containing $6^{13}C,2^{15}N$ (circled portion, delta 8), whereas the pre-derivatized internal standards are labeled with mass tag containing $6^{12}C,2^{14}N$ (circled portion, delta 0). The method performs primary and secondary amine derivatization **(B)**, affecting N-terminal and amine-containing functional groups. Stable-isotope coded reagents typically use internal standard addition after initial sample processing (e.g., following deproteinization, extraction, and derivatization) **(C)**, since the mass difference between endogenous analytes and standard used for quantification is based on isotopic differences within the derivatization reagent. Glycine is shown as an example. In contrast, the present method uses isotopically labeled IS addition as the first step of sample preparation **(D)**, providing broader internal standard coverage for the entire sample preparation.

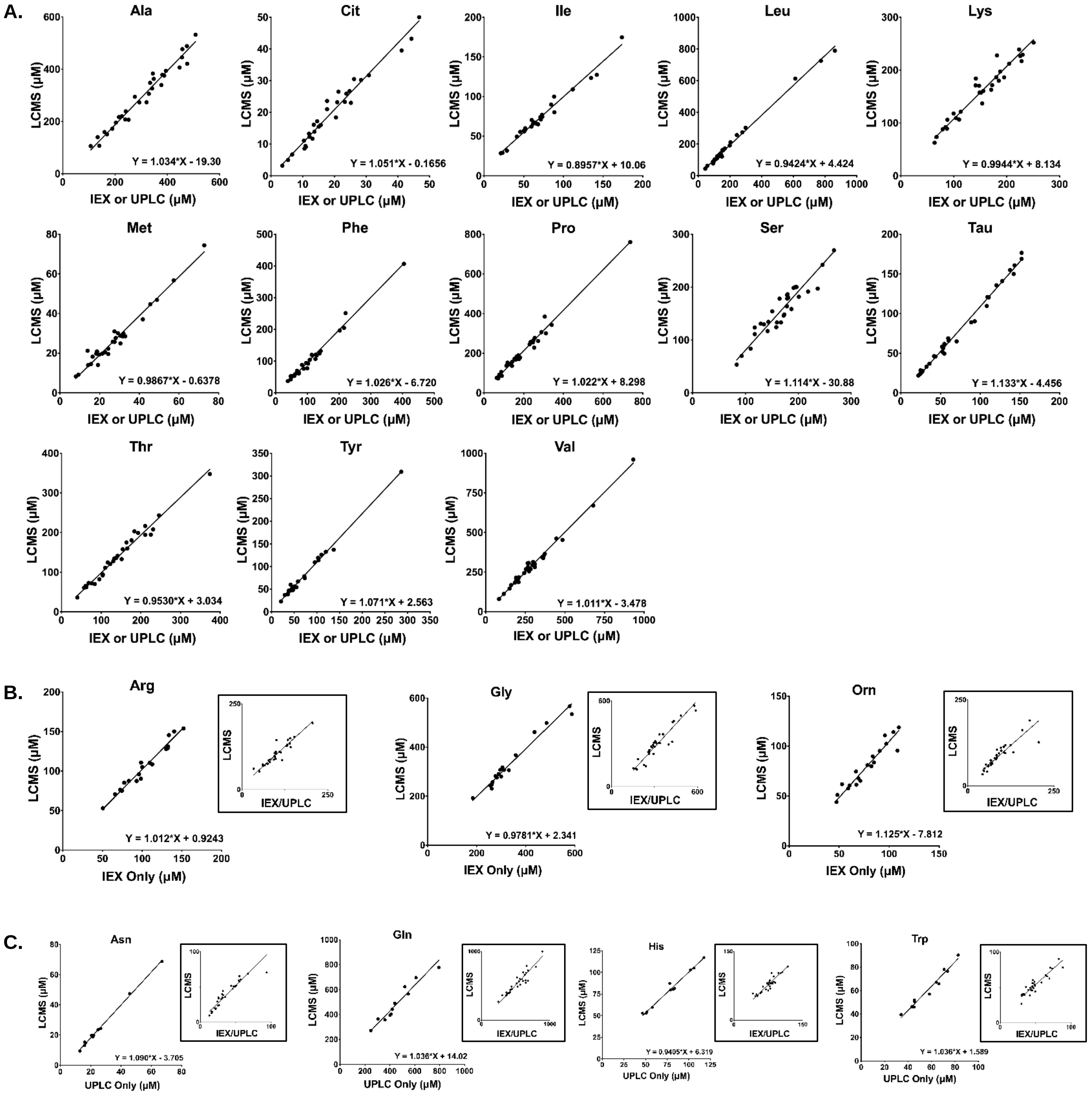

Figure S3. Method correlation. Fits based on Deming regression of the LCMS method and conventional testing (either IEX *or* UPLC) results are shown for 12 targets with R > 0.95 **(A)**. Similar level of correlation was seen for Arg, Gly, and Orn **(B)** when restricting analysis to IEX specimens and for Asn, Gln, His and Trp **(C)** when restricting analysis to UPLC (all specimens shown in inset).

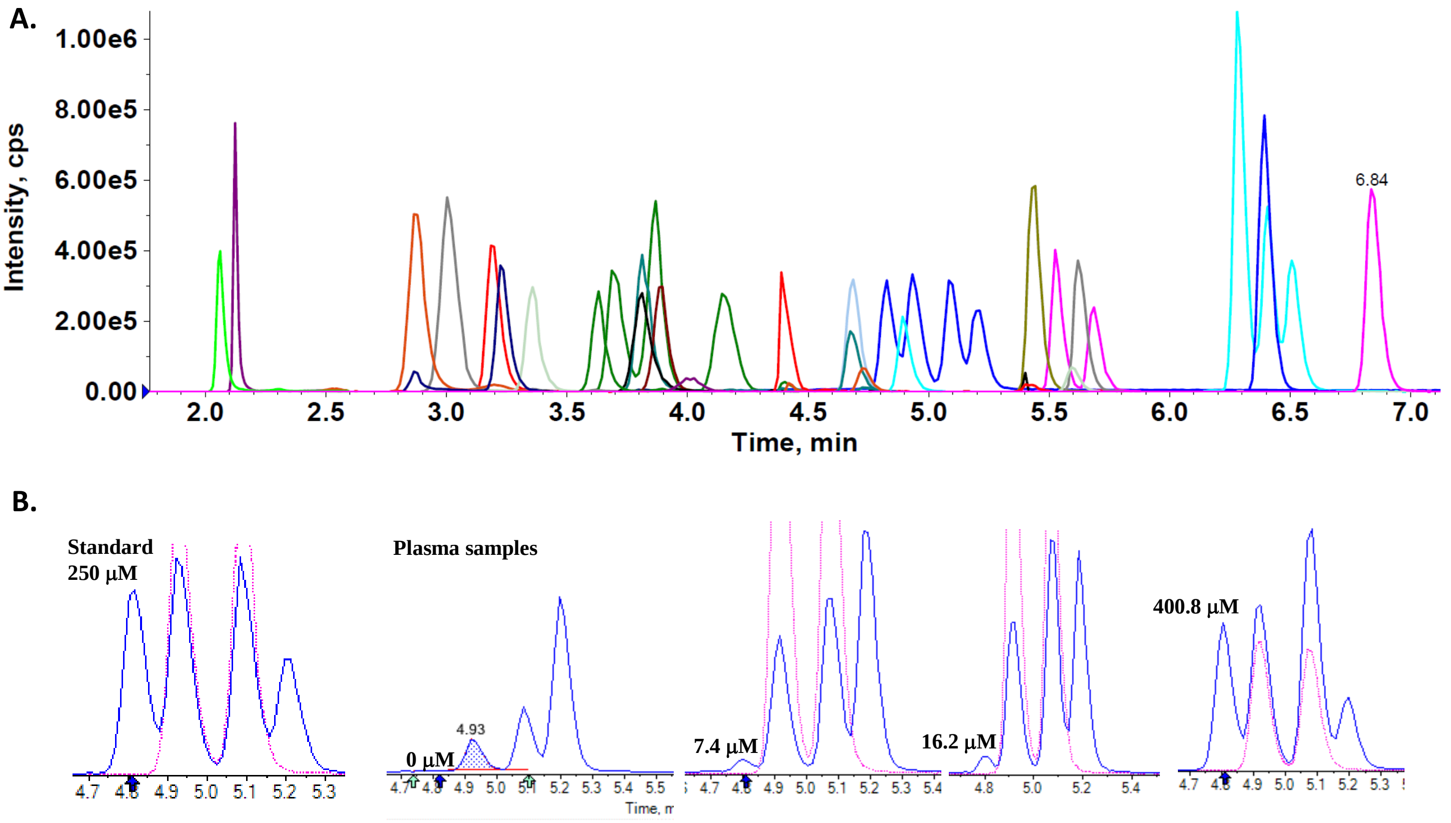

Figure S4. Representative ion chromatograms. An example total ion chromatogram (TIC) is shown for the external calibration standard **(A)**. Extracted ion chromatograms shown for the unlabeled leucine derivatives (blue peaks from left to right: *allo*-Ile, Ile, Leu, nor-Leu). The five plots, from left to right **(B)**, show 250 µM standard and patient plasmas containing 0, 7.4, 16.2, and 400.8 µM *allo*-Ile (values determined by ion-exchange chromatography).

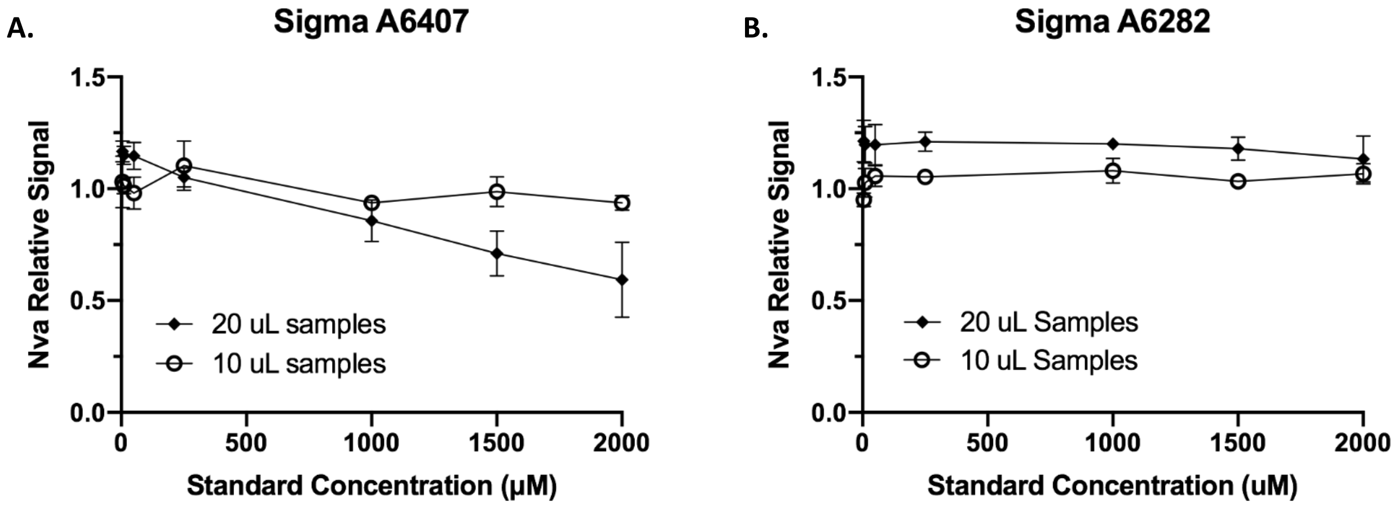

Figure S5. Relative norvaline signals in standards prepared from 5 to 2000 μM in 0.1 N HCl. Norvaline (Nva) is contained in the aTRAQ Labeling Buffer and functions as an internal standard in sample preparation from derivatization onward. The graphs show Nva signals (relative to Nva signal in a 250 μM external standard) in Sigma standards A6407 **(A)** and A6282 **(B)** prepared from 5 to 2000 μM in 0.1 N HCl. Because Nva it is added at an equal concentration across samples, one might expect the norvaline signal to remain constant across the standards. However, for the Sigma standard A6407 μL this was not the case when using 20 μL samples. A drop in Nva signal was observed in standards at increasing concentration. This effect was not observed for 10 μL samples, which effectively decreases the ratio of sample to derivatization reagent. Notably, the effect was also largely absent for standards prepared from A6282, which is unsurprising since A6407 contains 27 compounds whereas A6282 contains only 15. The effect could prevent accurate determination of ULOQ when using the A6407 standard, since the derivatization reagent is not in excess.
