# Supplementary material for: Quantitative amino acid analysis by liquid chromatography‐tandem mass spectrometry using low cost derivatization and an automated liquid handler
Source: JIMD Rep. 2019 Nov 12;51(1):62–9. doi: 10.1002/jmd2.12080 (PMC7012744; doi:10.1002/jmd2.12080)
Supplement: Supplementary file 2 — Table S1. Chromatography gradient Table S2. Selective reaction monitoring Table S3. Standard curve analysis—derivatized amino acids Table S4. Standard curve analysis—underivatized isoleucine and allo‐Ile Table S5. Supplementary precision data—derivatized amino acids Table S6. Supplementary precision data—underivatized isoleucine and allo‐Ile Table S7. Method correlation—patient plasma Table S8. Ion suppression/matrix effect data analysis Table S9. Spiked plasma analysis Table S10. Spiked urine analysis Table S11. Spiked CSF analysis Table S12. Unlabeled isoleucine and allo‐isoleucine in plasma Table S13. Matrix‐specific precision and signal suppression results Table S14. Lipemia mixing studies (plasma) [file JMD2-51-62-s002.pdf]

Supplementary Tables

Table of Contents

Table S1. Chromatography gradient..2

Table S2. Selective reaction monitoring……..…………………………….....……………………….. 3

Table S3. Standard curve analysis – derivatized amino acids ….…………………………..……………4

Table S4. Standard curve analysis – underivatized isoleucine and allo-Ile .....…………………......….... 10

Table S5. Supplementary precision data – derivatized amino acids.…………………………………….. 11

Table S6. Supplementary precision data – underivatized isoleucine and allo-Ile ………………………..12

Table S7. Method correlation – patient plasma ……………….....…………………..………………...13

Table S8. Ion Suppression/ Matrix Effect Data Analysis……….…………………………………..… 17

Table S9. Spiked plasma analysis…………..…………………..………………………………………18

Table S10. Spiked urine analysis……………………………………………………………………….19

Table S11. Spiked CSF analysis………………………………………………………………………..19

Table S12. Unlabeled isoleucine and allo-isoleucine in plasma………………………………….…… 19

Table S13. Matrix-specific precision and signal suppression results……………………………….....…20

Table S14. Lipemia mixing studies (plasma)…………………………………………………….…… 25

Table S1. Chromatography gradient.

Time (min)	Mobile Phase A (%) [a]	Mobile Phase B (%) [b]
0.00	97	3
4.40	60	40
6.50	60	40
6.80	10	90
7.50	10	90
7.80	97	3
11.00	97	3

[a] 0.1% formic acid and 0.01% heptafluorobutyric acid (v/v) in water
[b] 0.1% formic acid and 0.01% heptafluorobutyric acid (v/v) in methanol

Table S2. Selective Reaction Monitoring.

Target [a]	Q1/Q3 [b]	Time [c]	Window [d]	CE (eV)
PEA	290.1/121.1	2	60	33
Tau	274.1/121.1	2.14	40	30
Asn	281.2/121.1	2.88	50	30
Ser	254.2/121.1	3.02	50	27
Ser (I.S.)	258.2/121.1	3.02	50	27
Gly	224.1/121.1	3.2	50	25
Gly (I.S.)	227.2/121.1	3.2	50	25
Gln	295.2/121.1	3.24	50	30
Asp	282.1/121.1	3.37	50	29
Asp (I.S.)	287.2/121.1	3.37	50	29
His	304.2/121.1	3.7	60	28
His (I.S.)	313.2/121.1	3.7	60	28
Cit	324.2/121.1	3.55	50	30
Thr	268.2/121.1	3.7	50	28
Thr (I.S.)	273.2/121.1	3.9	50	28
Ala	238.2/121.1	3.8	50	30
Ala (I.S.)	242.2/121.1	3.8	50	30
Glu	296.2/121.1	3.75	50	27
Glu (I.S.)	302.2/121.1	3.75	50	27
ASA	439.2/121.1	3.8	65	40
Arg	323.2/121.1	4.3	60	32
Arg (I.S.)	333.2/121.1	4.3	60	32
HCT	338.2/121.1	4.32	50	28
Orn	429.3/121.1	4.5	50	40
Pro	264.2/121.1	4.6	40	37
Pro (I.S.)	270.2/121.1	4.6	40	37
Cys	537.2/121.1	4.65	40	50
Cys (I.S.)	545.3/121.1	4.65	40	50
Lys	443.3/121.1	4.8	40	40
Lys (I.S.)	451.3/121.1	4.8	40	40
uLeu deriv.	132.1/86.1	4.9	100	14
uLeu deriv. (I.S.)	139.1/92.1	4.9	100	14
Met (I.S.)	304.2/121.1	5.35	60	28
Met	298.2/121.1	5.35	60	28
Val	266.2/121.1	5.6	80	37
Val (I.S.)	272.2/121.1	5.6	80	37
Hcy	565.3/121.1	5.5	50	60
Tyr	330.2/121.1	5.62	40	30
Tyr (I.S.)	340.2/121.1	5.5	40	30
Ile/Leu	280.2/121.1	6.3	80	37
Ile/Leu (I.S.)	287.2/121.1	6.3	80	37
Phe	314.2/121.1	6.33	50	28
Phe (I.S.)	324.2/121.1	6.33	50	28
Trp	353.2/121.1	6.8	40	30

[a] Selective reaction monitoring targets consist of aTRAQ reagent (Δ8) derivatized amino acids and aTRAQ reagent (Δ8) derivatized stable-isotope (^{13}C,^{15}N) labeled AA analogs (I.S.), underivatized leucine derivatives, and underivatized isotopically labeled leucine derivatives, the latter two required for *allo*-Ile analysis and Ile measurement in the presence of allo-Ile. Phosphorylethanolamine is detectible but is not quantified or reported.

[b] Mass transitions (Q1/Q3) for target amino acids were provided by the kit manufacturer. Mass transitions for the analogous amino acid internal standards (^{13}C,^{15}N-labeled) were calculated based on the expected number of isotopically labeled carbon and nitrogen atoms in the native AA structure. Mass transition for underivatized, ^{13}C,^{15}N-labeled leucine derivatives ("uLeu deriv. (I.S.)") were determined empirically via direct infusion experiment.

[c] Chromatography peak center.

[d] Time window to include target as part of the SRM scanning, centered on the retention time.

Table S3. Standard curve analysis – derivatized amino acids (Page 1 of 6) .

Target		Run A		Run B		Run C		Analysis			Plot [a]
AA	Conc. (µM)	Result (µM)	Rec. (%)	Result (µM)	Rec. (%)	Result (µM)	Rec. (%)	Mean	SD	%CV	
**Ala**	5	5.3	106.2	3.8	76.7	4.6	92.4	4.6	0.74	16.02	
	10	10.8	107.7	10.0	99.7	10.0	100.1	10.2	0.45	4.43	
	50	46.1	92.1	48.5	97.0	48.4	96.7	47.6	1.36	2.85	
	250	250.4	100.2	220.3	88.1	235.9	94.4	235.5	15.09	6.41	
	1000	966.7	96.7	963.3	96.3	980.6	98.1	970.2	9.15	0.94	
	1500	1485.8	99.1	1460.0	97.3	1483.4	98.9	1476.4	14.25	0.97	
	2000	1930.2	96.5	1928.8	96.4	1829.1	91.5	1896.0	57.95	3.06	
**Arg**	5	4.2	84.9	5.3	106.5	5.7	113.2	5.1	0.74	14.61	
	10	10.1	100.5	9.0	90.1	10.2	102.0	9.8	0.65	6.64	
	50	47.4	94.7	47.1	94.2	46.7	93.4	47.1	0.33	0.71	
	250	241.3	96.5	225.1	90.0	233.7	93.5	233.4	8.09	3.47	
	1000	986.4	98.6	896.4	89.6	845.9	84.6	909.6	71.17	7.83	
	1500	1458.1	97.2	1316.4	87.8	1391.2	92.8	1388.6	70.93	5.11	
	2000	1809.4	90.5	1791.8	89.6	1770.2	88.5	1790.5	19.61	1.10	
**ASA**	5	5.8	115.7	8.1	161.7	5.94	118.8	6.6	1.29	19.48	
	10	8.7	87.1	11.5	115.3	11.69	116.9	10.6	1.68	15.75	
	50	55.1	110.2	50.0	100.0	46.08	92.2	50.4	4.52	8.97	
	250	289.8	115.9	265.3	106.1	256.99	102.8	270.7	17.05	6.30	
	1000	1164.2	116.4	1051.3	105.1	996.41	99.6	1070.6	85.55	7.99	
	1500	1661.9	110.8	1650.1	110.0	1599.10	106.6	1637.0	33.36	2.04	
	2000	2168.9	108.4	2119.0	106.0	2156.32	107.8	2148.1	25.92	1.21	
**Asn**	5	5.1	102.4	4.8	96.0	4.5	88.9	4.8	0.34	7.00	
	10	9.6	96.0	9.4	94.0	9.5	95.0	9.5	0.11	1.11	
	50	41.7	83.3	42.0	83.9	46.0	92.1	43.2	2.44	5.65	
	250	218.2	87.3	219.5	87.8	233.3	93.3	223.7	8.39	3.75	
	1000	911.4	91.1	904.6	90.5	894.4	89.4	903.4	8.54	0.95	
	1500	1326.8	88.5	1379.7	92.0	1257.9	83.9	1321.5	61.07	4.62	
	2000	1695.7	84.8	1766.9	88.4	1753.0	87.7	1738.5	37.78	2.17	
**Asp**	5	5.2	104.5	5.0	99.7	5.1	102.1	5.1	0.12	2.35	
	10	11.0	110.3	11.0	109.9	10.9	109.4	11.0	0.05	0.41	
	50	51.4	102.8	56.4	112.9	55.1	110.2	54.3	2.61	4.80	
	250	265.4	106.2	263.4	105.4	270.7	108.3	266.5	3.75	1.41	
	1000	1037.5	103.8	1117.7	111.8	1042.4	104.2	1065.9	44.96	4.22	
	1500	1604.4	107.0	1663.8	110.9	1570.4	104.7	1612.9	47.28	2.93	
	2000	2131.8	106.6	2088.8	104.4	2161.0	108.1	2127.2	36.35	1.71	

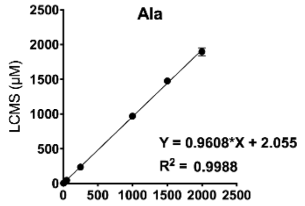

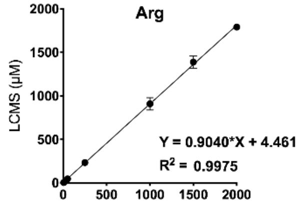

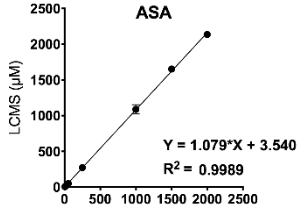

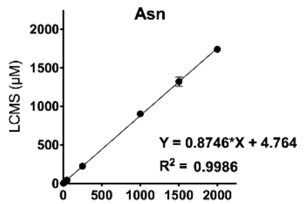

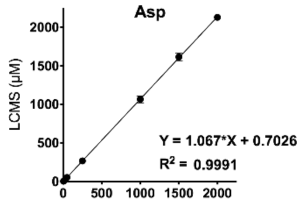

Dark green recovery (Rec.) cells indicate LCMS result within ±10% of expected target, light green within 20%, yellow within 30%, red >30% difference. ASA = argininosuccinic acid

[a] Target concentration on X-axis

Table S3. Standard curve analysis – derivatized amino acids (Page 2 of 6)

Target		Run A		Run B		Run C		Analysis			Plot [a]
AA	Conc. (µM)	Result (µM)	Rec. (%)	Result (µM)	Rec. (%)	Result (µM)	Rec. (%)	Mean	Std. Dev.	%CV	
Cit	5	4.5	90.4	4.8	94.9	4.5	90.7	4.6	0.13	2.83	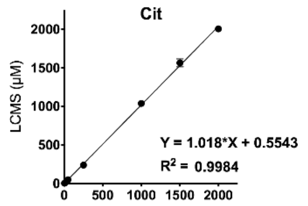
	10	8.5	85.0	9.4	94.2	9.7	96.8	9.2	0.62	6.74	
	50	49.5	99.0	48.3	96.7	49.7	99.5	49.2	0.75	1.53	
	250	239.5	95.8	226.1	90.4	252.9	101.2	239.5	13.41	5.60	
	1000	1007.8	100.8	1038.8	103.9	1068.0	106.8	1038.2	30.10	2.90	
	1500	1534.2	102.3	1532.2	102.2	1620.4	108.0	1562.3	50.33	3.22	
	2000	1971.7	98.6	1984.8	99.2	2052.5	102.6	2003.0	43.38	2.17	
Cys	5	2.3	92.0	2.1	83.7	2.3	90.6	2.2	0.11	5.12	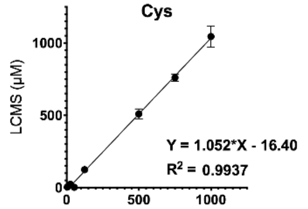
	10	4.1	81.5	4.8	94.9	5.5	109.2	4.8	0.69	14.49	
	50	23.4	93.5	23.9	95.5	24.2	96.7	23.8	0.40	1.67	
	250	122.2	97.7	128.6	102.9	122.9	98.3	124.6	3.51	2.82	
	1000	518.0	103.6	471.6	94.3	538.7	107.7	509.4	34.32	6.74	
	1500	744.4	99.3	747.7	99.7	789.3	105.3	760.5	25.07	3.30	
	2000	967.3	96.7	1057.0	105.7	1110.5	111.1	1044.9	72.36	6.93	
Gln	5	5.2	103.4	5.2	104.6	6.5	129.1	5.6	0.73	12.96	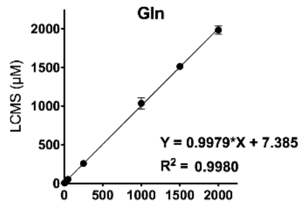
	10	10.5	105.1	11.0	109.9	10.5	105.3	10.7	0.27	2.54	
	50	53.0	105.9	54.4	108.7	51.4	102.9	52.9	1.48	2.79	
	250	261.0	104.4	264.4	105.8	246.2	98.5	257.2	9.70	3.77	
	1000	1092.5	109.3	1063.7	106.4	953.2	95.3	1036.5	73.51	7.09	
	1500	1550.1	103.3	1502.1	100.1	1481.9	98.8	1511.3	35.07	2.32	
	2000	2039.2	102.0	1971.7	98.6	1936.6	96.8	1982.5	52.13	2.63	
Glu	5	4.6	91.7	5.0	100.1	5.4	107.3	5.0	0.39	7.82	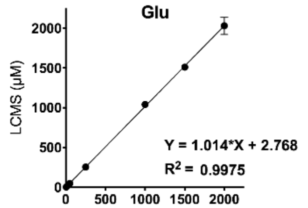
	10	10.2	102.1	10.4	103.5	10.2	102.0	10.3	0.08	0.82	
	50	47.9	95.7	52.2	104.4	51.7	103.4	50.6	2.37	4.69	
	250	256.4	102.6	260.6	104.3	251.1	100.4	256.0	4.80	1.87	
	1000	1042.2	104.2	1080.8	108.1	999.6	100.0	1040.9	40.63	3.90	
	1500	1548.5	103.2	1507.3	100.5	1474.4	98.3	1510.1	37.13	2.46	
	2000	1913.4	95.7	2051.9	102.6	2123.6	106.2	2029.6	106.87	5.27	
Gly	5	5.9	118.0	5.9	118.0	4.8	95.0	5.5	0.66	12.04	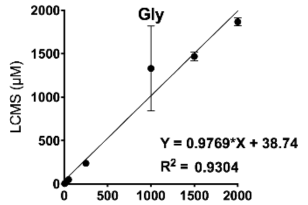
	10	11.6	116.3	11.0	110.4	10.9	108.7	11.2	0.40	3.57	
	50	48.7	97.4	52.4	104.7	50.0	100.0	50.3	1.87	3.72	
	250	242.3	96.9	239.3	95.7	238.2	95.3	240.0	2.13	0.89	
	1000	1041.7	104.2	1055.9	105.6	1896.9	189.7	1331.5	489.72	36.78	
	1500	1528.0	101.9	1434.9	95.7	1443.2	96.2	1468.7	51.51	3.51	
	2000	1858.1	92.9	1915.2	95.8	1829.9	91.5	1867.8	43.48	2.33	

Dark green recovery (Rec.) cells indicate LCMS result within ±10% of expected target, light green within 20%, yellow within 30%, red >30% difference.

[a] Target concentration on X-axis

Table S3. Standard curve analysis – derivatized amino acids (Page 3 of 6)

Target		Run A		Run B		Run C		Analysis			Plot [a]
AA	Conc. (µM)	Result (µM)	Rec. (%)	Result (µM)	Rec. (%)	Result (µM)	Rec. (%)	Mean	SD	%CV	
**HCY**	5	7.0	139.3	5.6	112.7	4.8	95.3	5.8	1.10	19.07	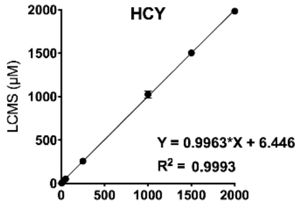
	10	11.7	116.7	10.9	108.8	10.8	108.2	11.1	0.47	4.26	
	50	56.5	113.0	50.1	100.3	51.5	103.0	52.7	3.36	6.37	
	250	274.7	109.9	237.6	95.1	260.4	104.2	257.6	18.69	7.26	
	1000	1047.4	104.7	980.4	98.0	1050.5	105.1	1026.1	39.58	3.86	
	1500	1523.0	101.5	1493.2	99.6	1496.4	99.8	1504.2	16.37	1.09	
	2000	1968.0	98.4	1975.9	98.8	2010.1	100.5	1984.6	22.42	1.13	
**His**	5	4.6	92.9	4.4	87.6	5.2	104.0	4.7	0.42	8.84	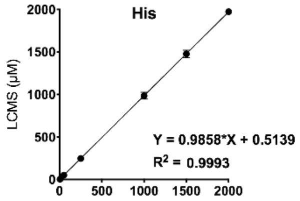
	10	9.9	99.0	9.4	94.2	10.6	106.4	10.0	0.61	6.15	
	50	52.8	105.6	50.1	100.2	50.7	101.4	51.2	1.43	2.78	
	250	247.2	98.9	247.1	98.8	248.0	99.2	247.4	0.53	0.21	
	1000	982.0	98.2	947.3	94.7	1027.7	102.8	985.7	40.32	4.09	
	1500	1526.1	101.7	1465.9	97.7	1440.6	96.0	1477.5	43.92	2.97	
	2000	1960.2	98.0	1990.8	99.5	1969.5	98.5	1973.5	15.69	0.80	
**Ile**	5	4.5	90.9	4.7	93.8	4.7	93.8	4.6	0.09	1.87	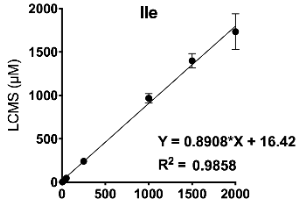
	10	10.4	103.5	9.2	92.0	10.0	100.3	9.9	0.59	6.02	
	50	52.5	104.9	46.3	92.6	46.0	92.0	48.3	3.65	7.57	
	250	249.7	99.9	242.9	97.2	232.5	93.0	241.7	8.68	3.59	
	1000	1017.6	101.8	910.2	91.0	975.3	97.5	967.7	54.10	5.59	
	1500	1482.5	98.8	1316.0	87.7	1397.6	93.2	1398.7	83.27	5.95	
	2000	1971.1	98.6	1599.3	80.0	1630.0	81.5	1733.5	206.38	11.91	
**Leu**	5	3.3	66.6	5.3	105.4	4.5	90.1	4.4	0.98	22.37	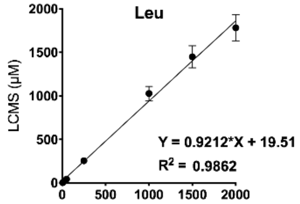
	10	8.4	83.6	8.8	87.8	8.6	86.0	8.6	0.21	2.46	
	50	43.3	86.7	48.4	96.8	42.9	85.8	44.9	3.07	6.84	
	250	258.1	103.2	272.4	108.9	235.3	94.1	255.3	18.67	7.31	
	1000	976.8	97.7	1121.1	112.1	983.2	98.3	1027.0	81.53	7.94	
	1500	1390.1	92.7	1594.3	106.3	1363.1	90.9	1449.1	126.40	8.72	
	2000	1712.8	85.6	1955.6	97.8	1680.0	84.0	1782.8	150.54	8.44	
**Lys**	5	5.6	112.8	5.3	106.4	5.9	118.3	5.6	0.30	5.34	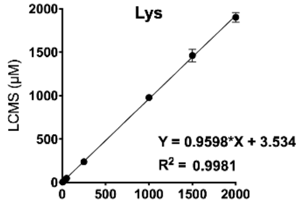
	10	11.4	114.0	10.3	102.9	10.9	109.4	10.9	0.56	5.13	
	50	50.5	101.1	49.5	99.0	47.2	94.4	49.1	1.70	3.47	
	250	244.7	97.9	234.9	94.0	236.2	94.5	238.6	5.32	2.23	
	1000	1001.9	100.2	986.6	98.7	942.0	94.2	976.8	31.14	3.19	
	1500	1537.8	102.5	1395.3	93.0	1454.2	97.0	1462.5	71.57	4.89	
	2000	1953.7	97.7	1845.2	92.3	1908.6	95.4	1902.5	54.50	2.86	

Dark green recovery (Rec.) cells indicate LCMS result within ±10% of expected target, light green within 20%, yellow within 30%, red >30% difference. HCY = homocystine

[a] Target concentration on X-axis

Table S3. Standard curve analysis – derivatized amino acids (Page 4 of 6)

Target		Run A		Run B		Run C		Analysis			
AA	Conc. (µM)	Result (µM)	Rec. (%)	Result (µM)	Rec. (%)	Result (µM)	Rec. (%)	Mean	SD	% CV	Plot [a]
Met	5	4.7	93.5	4.6	91.9	4.6	91.3	4.6	0.06	1.32	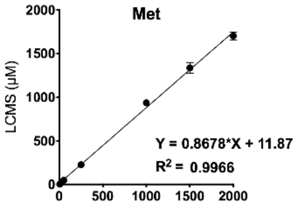
	10	9.6	95.5	9.4	94.0	9.3	93.3	9.4	0.11	1.19	
	50	50.2	100.3	46.9	93.7	43.3	86.5	46.8	3.45	7.37	
	250	224.3	89.7	230.4	92.2	221.9	88.8	225.5	4.39	1.95	
	1000	943.4	94.3	950.8	95.1	913.5	91.4	935.9	19.72	2.11	
	1500	1397.8	93.2	1274.5	85.0	1336.3	89.1	1336.2	61.62	4.61	
	2000	1703.8	85.2	1657.2	82.9	1748.0	87.4	1703.0	45.43	2.67	
Orn	5	4.8	96.1	4.9	97.7	5.3	106.2	5.0	0.27	5.49	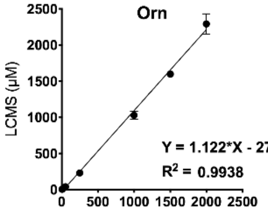
	10	9.5	94.5	8.2	81.7	9.2	92.0	8.9	0.68	7.59	
	50	39.2	78.4	44.2	88.4	41.5	83.1	41.6	2.51	6.03	
	250	251.5	100.6	217.7	87.1	230.0	92.0	233.0	17.14	7.35	
	1000	1090.3	109.0	1011.2	101.1	987.0	98.7	1029.5	54.02	5.25	
	1500	1628.6	108.6	1622.7	108.2	1547.4	103.2	1599.6	45.28	2.83	
	2000	2411.6	120.6	2137.5	106.9	2326.5	116.3	2291.9	140.30	6.12	
Phe	5	4.9	97.8	4.9	98.1	4.6	92.2	4.8	0.16	3.43	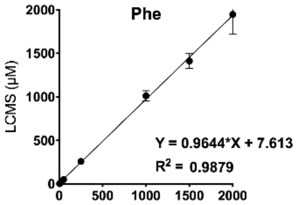
	10	10.8	107.6	10.2	102.2	10.1	100.8	10.4	0.36	3.47	
	50	52.7	105.4	50.5	101.1	49.2	98.4	50.8	1.77	3.49	
	250	265.7	106.3	269.3	107.7	244.3	97.7	259.7	13.52	5.21	
	1000	1065.7	106.6	1021.3	102.1	951.1	95.1	1012.7	57.78	5.71	
	1500	1507.0	100.5	1339.8	89.3	1389.0	92.6	1411.9	85.95	6.09	
	2000	2170.6	108.5	1715.7	85.8	1953.8	97.7	1946.7	227.54	11.69	
Pro	5	4.54	90.7	4.7	93.9	5.2	103.1	4.8	0.32	6.74	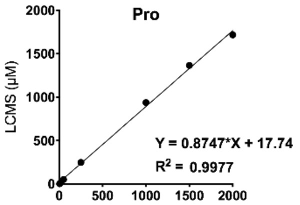
	10	10.51	105.1	10.7	107.1	10.5	104.8	10.6	0.13	1.18	
	50	52.65	105.3	52.4	104.9	52.0	103.9	52.3	0.35	0.67	
	250	246.72	98.7	242.0	96.8	257.3	102.9	248.7	7.83	3.15	
	1000	954.15	95.4	919.0	91.9	938.6	93.9	937.3	17.64	1.88	
	1500	1361.75	90.8	1367.3	91.2	1366.8	91.1	1365.3	3.06	0.22	
	2000	1709.16	85.5	1743.3	87.2	1698.2	84.9	1716.9	23.53	1.37	
Ser	5	6.0	119.6	5.2	103.2	5.1	102.2	5.4	0.49	9.02	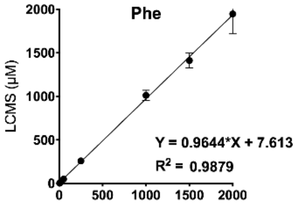
	10	11.0	110.3	10.3	103.4	10.4	103.5	10.6	0.40	3.74	
	50	51.2	102.4	53.4	106.8	49.1	98.1	51.2	2.17	4.23	
	250	256.0	102.4	232.1	92.8	252.9	101.2	247.0	13.01	5.27	
	1000	989.5	99.0	1005.5	100.6	994.7	99.5	996.5	8.15	0.82	
	1500	1495.1	99.7	1421.3	94.8	1444.4	96.3	1453.6	37.79	2.60	
	2000	1874.1	93.7	1710.6	85.5	1860.4	93.0	1815.1	90.71	5.00	

Dark green recovery (Rec.) cells indicate LCMS result within ±10% of expected target, light green within 20%, yellow within 30%, red >30% difference.

[a] Target concentration on X-axis

Table S3. Standard curve analysis – derivatized amino acids (Page 5 of 6)

Target		Run A		Run B		Run C		Analysis			Plot [a]
AA	Conc. (µM)	Result (µM)	Rec. (%)	Result (µM)	Rec. (%)	Result (µM)	Rec. (%)	Mean	SD	%CV	
Tau	5	4.6	92.7	5.1	102.8	5.2	103.5	5.0	0.30	6.03	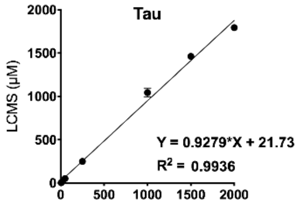
	10	10.2	102.3	10.2	101.8	10.6	105.8	10.3	0.22	2.11	
	50	54.1	108.2	51.7	103.5	52.4	104.9	52.7	1.20	2.27	
	250	251.5	100.6	251.6	100.6	247.9	99.2	250.3	2.10	0.84	
	1000	1070.1	107.0	1076.0	107.6	987.9	98.8	1044.7	49.26	4.72	
	1500	1499.0	99.9	1427.3	95.2	1462.1	97.5	1462.8	35.84	2.45	
	2000	1784.2	89.2	1800.8	90.0	1796.7	89.8	1793.9	8.66	0.48	
Thr	5	4.0	79.4	3.7	73.0	3.8	75.7	3.8	0.16	4.24	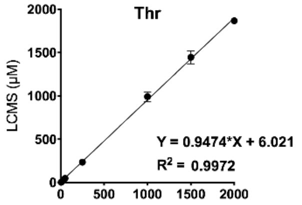
	10	8.1	81.0	9.4	94.2	9.1	91.2	8.9	0.69	7.79	
	50	48.4	96.8	48.0	96.1	49.4	98.8	48.6	0.71	1.47	
	250	231.8	92.7	244.8	97.9	231.2	92.5	236.0	7.70	3.26	
	1000	999.1	99.9	1044.6	104.5	933.9	93.4	992.5	55.64	5.61	
	1500	1475.4	98.4	1501.4	100.1	1360.9	90.7	1445.9	74.71	5.17	
	2000	1847.9	92.4	1912.9	95.6	1844.0	92.2	1868.3	38.68	2.07	
Trp	5	4.7	94.3	4.8	95.3	4.7	94.0	4.7	0.03	0.65	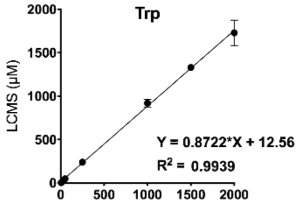
	10	9.6	96.1	9.9	98.8	9.3	92.8	9.6	0.30	3.13	
	50	51.2	102.5	48.5	96.9	48.2	96.5	49.3	1.68	3.40	
	250	244.9	98.0	249.8	99.9	229.9	91.9	241.5	10.38	4.30	
	1000	958.5	95.9	933.8	93.4	871.5	87.2	921.3	44.87	4.87	
	1500	1350.9	90.1	1334.9	89.0	1308.6	87.2	1331.5	21.38	1.61	
	2000	1897.1	94.9	1669.2	83.5	1622.4	81.1	1729.5	146.98	8.50	
Tyr	5	4.3	85.9	5.2	104.1	4.9	96.9	4.8	0.45	9.48	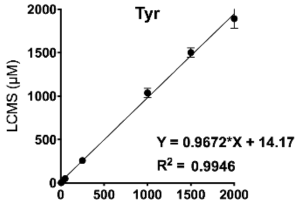
	10	9.5	95.2	10.2	101.9	9.8	97.5	9.8	0.34	3.47	
	50	50.0	100.0	52.1	104.2	50.0	99.9	50.7	1.22	2.40	
	250	258.7	103.5	265.1	106.1	251.7	100.7	258.5	6.74	2.61	
	1000	1009.6	101.0	1099.4	109.9	1003.8	100.4	1037.6	53.57	5.16	
	1500	1559.4	104.0	1495.7	99.7	1452.3	96.8	1502.5	53.89	3.59	
	2000	1955.3	97.8	1956.6	97.8	1765.7	88.3	1892.5	109.86	5.80	
Val	5	4.0	80.6	3.4	68.5	4.0	79.3	3.8	0.33	8.67	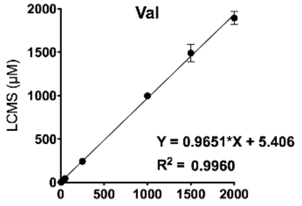
	10	8.7	87.1	8.3	83.4	8.5	85.4	8.5	0.19	2.17	
	50	45.0	89.9	45.6	91.1	44.7	89.5	45.1	0.42	0.93	
	250	252.7	101.1	239.9	96.0	242.0	96.8	244.9	6.84	2.79	
	1000	1027.7	102.8	978.8	97.9	985.3	98.5	997.3	26.58	2.67	
	1500	1592.6	106.2	1486.6	99.1	1394.1	92.9	1491.1	99.34	6.66	
	2000	1932.5	96.6	1942.4	97.1	1808.0	90.4	1894.3	74.91	3.95	

Dark green recovery (Rec.) cells indicate LCMS result within ±10% of expected target, light green within 20%, yellow within 30%, red >30% difference.
[a] Target concentration on X-axis

Table S3. Standard curve analysis – derivatized amino acids (Page 6 of 6)

Target		Run A		Run B		Run C		Analysis			Plot [a]
AA	Conc. (µM)	Result (µM)	Rec. (%)	Result (µM)	Rec. (%)	Result (µM)	Rec. (%)	Mean	SD	%CV	
HCT	5	5.3	105.0	5.9	117.9	5.7	113.5	5.6	0.33	5.88	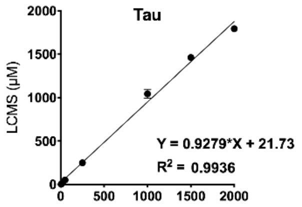
	10	9.6	96.0	10.7	107.1	10.9	108.7	10.4	0.69	6.66	
	50	46.9	93.8	50.2	100.5	54.3	108.6	50.5	3.70	7.33	
	250	229.3	91.7	250.2	100.1	267.3	106.9	248.9	19.04	7.65	
	1000	926.1	92.6	938.7	93.9	1090.4	109.0	985.0	91.47	9.29	
	1500	1436.7	95.8	1413.4	94.2	1650.4	110.0	1500.2	130.65	8.71	
	2000	1952.7	97.6	1923.1	96.2	2138.5	106.9	2004.8	116.74	5.82	

Dark green recovery (Rec.) cells indicate LCMS result within ±10% of expected target, light green within 20%, yellow within 30%, red >30% difference. HCT = homocitrulline

[a] Target concentration on X-axis

Table S4. Standard curve analysis – underivatized isoleucine and *allo*-Ile.

Std #	STD Concentrations		LCMS Results			Recovery (%)		Inter-Run Statistics [a]		
	[ILE]	[allo]	Run	[Ile]	[allo]	Ile	allo	$\bar{x}$	SD	%CV
			1	474.0	5.4	94.8	108.4			
1	500	5	2	486.5	4.9	97.3	97.6	4.9	0.5	**10.2**
			3	479.3	4.4	95.9	88.5			
			1	457.3	9.8	91.5	97.9			
2	500	10	2	482.0	8.6	96.4	85.8	9.1	0.6	**6.7**
			3	489.8	9.1	98.0	90.6			
			1	462.9	46.8	92.6	93.7			
3	500	50	2	497.9	53.1	99.6	106.2	48.6	3.9	**8.1**
			3	492.2	45.8	98.4	91.7			
			1	464.0	101.3	92.8	101.3			
4	500	100	2	495.7	108.4	99.1	108.4	102.9	4.8	**4.7**
			3	473.9	99.1	94.8	99.1			
			1	484.4	517.8	96.9	103.6			
5	500	500	2	509.4	511.2	101.9	102.3	520.0	10.1	**1.9**
			3	477.1	531.0	95.4	106.2			
			1	6.4	10.4	127.7	103.7			
6	5	10	2	7.2	10.5	144.9	104.9	7.0	0.6	**8.0**
			3	7.5	10.0	149.0	100.0			
			1	24.4	10.9	97.8	109.4			
7	25	10	2	25.5	10.6	102.2	105.9	25.7	1.3	**5.2**
			3	27.1	10.9	108.4	109.3			
			1	468.0	10.1	93.6	101.0			
8	500	10	2	487.0	8.5	97.4	85.2	484.5	15.4	**3.2**
			3	498.5	8.2	99.7	81.8			
			1	909.7	10.0	91.0	99.9			
9	1000	10	2	954.0	8.9	95.4	88.8	934.9	22.8	**2.4**
			3	941.0	9.2	94.1	91.6			
			1	1574.8	10.1	78.7	100.6			
10	2000	10	2	1687.2	10.0	84.4	99.7	1681.7	104.3	**6.2**
			3	1783.2	9.7	89.2	97.0			

Plots

All Points

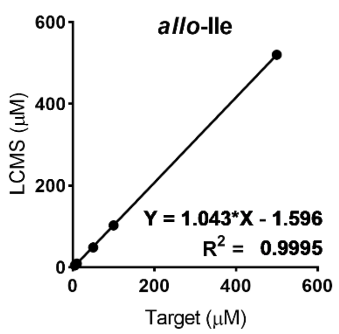

Low Concentrations

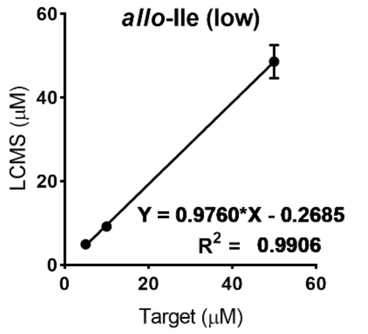

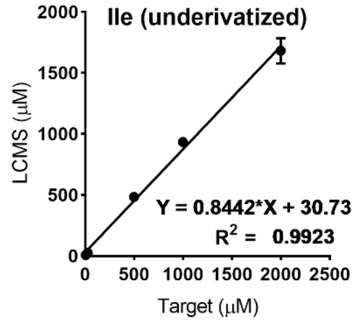

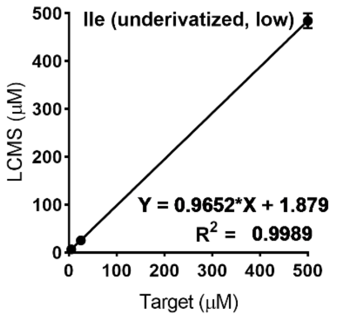

[a] Statistics apply to *allo*-Ile for samples 1 to 5, and apply to Ile for samples 6 to 10.

Table S5. Supplementary precision data – derivatized amino acids (control samples and plasma)

Interrun (Low)

#	Ala	Arg	ASA	Asn	Asp	Cit	Cys	Glu	Gly	Gln	HCY	His	Ile	Leu	Lys	Met	Orn	Phe	Pro	Ser	Tau	Thr	Trp	Tyr	Val
1	48.5	45.4	81.0	47.7	45.7	47.5	45.8	49.9	49.8	52.1	49.6	49.0	46.7	46.5	50.2	41.1	45.9	50.0	47.5	48.4	49.4	43.2	44.8	45.8	43.6
2	45.9	43.8	73.9	46.0	48.0	46.0	47.6	50.6	48.2	48.1	50.4	47.9	46.2	41.3	47.2	45.7	47.6	51.5	50.9	52.3	47.6	46.3	44.6	46.6	41.4
3	53.4	47.8	82.5	47.9	50.2	50.4	48.6	51.9	49.9	54.4	49.8	53.4	51.3	45.9	47.9	50.3	43.1	51.5	57.9	51.2	49.7	49.0	50.0	47.4	43.8
4	50.3	51.9	78.9	49.4	52.8	50.2	47.4	47.5	53.2	48.9	55.6	52.9	48.8	52.8	47.4	46.0	38.1	52.2	49.5	47.1	50.8	46.7	51.0	46.5	42.0
5	47.2	43.8	74.7	48.1	47.3	43.8	48.2	48.8	44.3	49.2	50.4	50.3	43.5	46.4	48.5	43.4	45.3	51.9	46.2	45.6	46.9	46.5	48.3	46.1	40.1
6	42.2	46.4	82.4	46.6	44.6	46.2	51.6	50.6	44.8	52.6	56.2	54.6	48.7	42.3	48.1	42.1	41.0	46.6	49.0	47.0	47.0	41.2	44.1	46.1	43.2
7	47.5	43.2	75.5	45.8	51.5	48.6	47.3	50.5	54.1	49.6	51.6	44.9	49.6	46.4	47.4	47.3	46.1	49.0	54.6	46.5	50.2	45.6	48.1	47.4	43.5
8	46.0	47.2	84.6	46.3	45.9	45.5	44.2	47.7	46.4	44.4	50.2	44.9	44.5	42.5	44.9	40.5	39.9	44.9	48.1	46.7	43.9	43.9	45.3	44.4	41.0
9	49.0	42.3	NP	48.6	50.7	46.2	49.5	51.5	49.2	NP	50.7	47.0	49.2	42.3	49.1	40.4	43.4	47.2	47.0	48.2	41.6	45.8	44.9	43.7	42.3
10	44.5	47.5	NP	49.6	36.7	35.5	45.8	47.6	44.8	NP	42.2	41.0	50.3	51.2	54.9	45.6	39.5	44.0	43.3	42.2	47.3	40.0	36.5	39.1	36.2
$\bar{x}$	47.4	45.9	79.2	47.6	47.3	46.0	47.6	49.7	48.5	49.9	50.7	48.6	47.9	45.8	48.5	44.3	43.0	48.9	49.4	47.5	47.4	44.8	45.8	45.3	41.7
SD	3.1	2.9	4.1	1.4	4.6	4.2	2.1	1.6	3.4	3.1	3.8	4.3	2.6	3.9	2.6	3.3	3.2	3.0	4.2	2.8	2.9	2.7	4.1	2.5	2.3
%CV	6.6	6.2	5.2	2.9	9.8	9.2	4.3	3.3	7.1	6.2	7.5	8.9	5.4	8.5	5.4	7.4	7.5	6.2	8.5	5.9	6.1	6.1	8.9	5.4	5.5

Interrun (High)

#	Ala	Arg	ASA	Asn	Asp	Cit	Cys	Glu	Gly	Gln	HCY	His	Ile	Leu	Lys	Met	Orn	Phe	Pro	Ser	Tau	Thr	Trp	Tyr	Val
1	244.8	223.5	794.6	264.1	260.2	254.2	244.8	245.0	240.0	542.8	241.3	240.2	230.5	240.5	254.8	214.4	256.4	257.0	**247.7**	243.8	261.0	254.3	246.3	244.8	226.1
2	256.8	231.1	761.0	253.3	255.2	238.5	252.6	265.7	256.2	514.6	278.9	255.8	262.6	246.7	244.4	231.3	249.0	271.9	236.7	260.9	241.5	241.7	246.0	263.7	237.3
3	261.8	231.7	786.8	246.3	246.2	240.1	223.7	243.1	242.1	527.2	259.7	234.7	270.8	275.7	225.0	228.0	236.4	256.9	236.5	242.7	234.5	250.5	245.1	248.6	227.8
4	256.6	247.3	787.6	247.3	249.3	258.1	242.3	244.9	276.0	497.1	257.7	249.9	252.1	260.2	243.6	241.9	212.5	270.4	240.4	236.2	240.4	231.3	252.0	249.4	216.1
5	261.2	235.8	757.1	249.4	257.4	222.4	232.0	255.7	241.4	531.4	227.2	240.9	241.6	267.3	244.3	230.8	229.3	267.7	227.2	251.1	250.1	237.3	255.1	251.7	234.9
6	240.4	244.2	702.7	258.3	229.9	242.2	232.6	246.4	253.9	425.9	240.8	233.6	262.1	235.3	246.5	240.4	213.3	243.1	232.1	256.5	233.6	233.9	242.4	243.4	231.1
7	255.0	246.1	762.3	253.3	277.8	254.9	227.1	243.3	267.8	453.4	253.4	235.9	266.4	252.5	257.8	238.2	263.0	257.0	263.7	242.7	233.7	237.4	256.7	238.1	236.5
8	250.3	224.3	710.4	247.0	248.8	239.0	251.2	246.1	240.0	538.3	248.8	235.9	238.1	230.6	235.4	223.7	267.9	244.7	241.9	247.7	222.1	242.6	239.8	233.1	228.0
9	255.4	245.4	NP	252.3	254.7	241.1	242.2	261.3	242.1	NP	240.1	244.0	252.3	252.8	251.8	215.3	243.6	263.5	240.3	233.7	222.4	242.3	232.4	217.0	222.9
10	234.9	260.3	NP	245.4	255.1	232.3	233.9	233.2	231.1	NP	216.7	232.0	273.6	249.3	260.5	218.4	238.1	236.5	238.4	241.8	203.0	223.0	230.3	228.7	244.4
$\bar{x}$	251.7	239.0	757.8	251.7	253.4	242.3	238.2	248.5	249.1	503.8	246.5	240.3	255.0	251.1	246.4	228.2	240.9	256.9	240.5	245.7	234.2	239.4	244.6	241.8	230.5
SD	9.0	11.7	34.6	5.9	12.1	10.9	9.9	9.7	14.1	42.8	17.5	7.7	14.6	14.0	10.7	10.2	19.0	12.1	9.9	8.5	16.1	9.1	8.8	13.2	8.1
%CV	3.6	4.9	4.6	2.4	4.8	4.5	4.1	3.9	5.7	8.5	7.1	3.2	5.7	5.6	4.3	4.4	7.9	4.7	4.1	3.5	6.9	3.8	3.6	5.5	3.5

Interrun (Plasma)

#	Ala	Arg	ASA	Asn	Asp	Cit	Cys	Glu	Gly	Gln	HCY	His	Ile	Leu	Lys	Met	Orn	Phe	Pro	Ser	Tau	Thr	Trp	Tyr	Val
1	217.2	98.4	NP	19.8	24.1	13.3	40.4	52.1	367.3	622.8	NP	87.3	55.4	101.2	170.4	31.0	105.2	119.9	152.5	129.6	28.3	175.3	51.9	59.9	305.8
2	241.5	104.3	NP	23.8	29.5	14.3	39.6	59.3	310.4	617.0	NP	84.2	54.2	110.3	177.9	31.9	109.1	118.4	127.0	148.7	30.5	184.0	51.3	61.2	300.0
3	240.1	115.9	NP	25.7	33.4	14.0	34.4	52.1	299.2	567.5	NP	88.6	61.2	99.2	169.5	29.4	98.4	124.9	133.2	129.6	26.3	178.9	47.1	59.9	300.5
$\bar{x}$	232.9	106.2	NP	23.1	29.0	13.9	38.1	54.5	325.6	602.4	NP	86.7	56.9	103.6	172.6	30.8	104.2	121.1	137.6	136.0	28.4	179.4	50.1	60.4	302.1
SD	13.6	8.9	NP	3.0	4.7	0.5	3.3	4.2	36.5	30.4	NP	2.2	3.7	5.9	4.6	1.3	5.5	3.4	13.3	11.0	2.1	4.4	2.6	0.8	3.2
%CV	5.8	8.4	NP	13.0	16.2	3.7	8.5	7.7	11.2	5.0	NP	2.6	6.5	5.7	2.7	4.1	5.2	2.8	9.7	8.1	7.4	2.4	5.2	1.3	1.1

Intrarun (Low)

#	Ala	Arg	ASA	Asn	Asp	Cit	Cys	Glu	Gly	Gln	HCY	His	Ile	Leu	Lys	Met	Orn	Phe	Pro	Ser	Tau	Thr	Trp	Tyr	Val
1	53.4	47.8	74.7	47.9	50.2	50.4	48.6	51.9	49.9	49.2	49.8	53.4	51.3	45.9	47.9	50.3	43.1	51.5	57.9	51.2	49.7	49.0	50.0	47.4	43.8
2	55.3	46.8	72.8	51.3	48.7	49.6	47.2	48.6	48.3	48.3	51.0	49.2	50.6	51.6	48.5	43.8	43.5	52.7	55.7	50.7	51.3	50.0	51.8	50.0	43.8
3	53.8	43.3	73.6	50.0	49.2	48.2	46.1	47.3	48.3	46.6	48.3	50.8	51.4	55.2	46.4	46.0	44.7	52.7	50.7	50.4	51.1	43.6	47.7	46.0	44.2
$\bar{x}$	54.2	46.0	73.7	49.7	49.3	49.4	47.3	49.3	48.8	48.0	49.7	51.1	51.1	50.9	47.6	46.7	43.8	52.3	54.8	50.8	50.7	47.5	49.8	47.8	43.9
SD	1.0	2.4	1.0	1.7	0.7	1.1	1.2	2.4	0.9	1.3	1.3	2.1	0.4	4.7	1.1	3.3	0.8	0.7	3.7	0.4	0.9	3.4	2.0	2.0	0.2
%CV	1.8	5.1	1.3	3.4	1.5	2.2	2.6	4.8	1.9	2.8	2.7	4.2	0.8	9.3	2.3	7.1	1.9	1.3	6.7	0.8	1.7	7.2	4.1	4.3	0.5

Intrarun (High)

#	Ala	Arg	ASA	Asn	Asp	Cit	Cys	Glu	Gly	Gln	HCY	His	Ile	Leu	Lys	Met	Orn	Phe	Pro	Ser	Tau	Thr	Trp	Tyr	Val
1	240.4	244.2	761.0	258.3	229.9	242.2	232.6	246.4	253.9	514.6	240.8	233.6	262.1	235.3	254.9	240.4	213.3	243.1	232.1	256.5	233.6	233.9	242.4	243.4	231.1
2	242.6	275.6	811.8	249.2	251.0	237.3	242.7	261.1	248.2	504.2	245.7	248.8	265.6	249.9	245.6	232.0	270.5	256.4	243.5	275.1	238.9	229.4	242.7	247.4	234.8
3	244.7	244.1	829.6	261.3	237.6	272.2	237.5	265.7	254.9	536.1	250.4	242.4	258.5	230.5	245.6	239.4	218.2	249.9	238.1	252.4	248.4	234.6	254.0	235.3	229.2
$\bar{x}$	242.5	254.6	800.8	256.3	239.5	250.6	237.6	257.7	252.3	518.3	245.6	241.6	262.0	238.6	248.7	237.3	234.0	249.8	237.9	261.3	240.3	232.6	246.4	242.0	231.7
SD	2.1	18.2	35.6	6.3	10.6	18.9	5.0	10.1	3.6	16.3	4.8	7.6	3.6	10.1	5.4	4.6	31.7	6.7	5.7	12.1	7.5	2.8	6.6	6.2	2.9
%CV	0.9	7.1	4.4	2.5	4.4	7.6	2.1	3.9	1.4	3.1	2.0	3.1	1.4	4.2	2.2	1.9	13.5	2.7	2.4	4.6	3.1	1.2	2.7	2.5	1.2

Intrarun (Plasma)

#	Ala	Arg	ASA	Asn	Asp	Cit	Cys	Glu	Gly	Gln	HCY	His	Ile	Leu	Lys	Met	Orn	Phe	Pro	Ser	Tau	Thr	Trp	Tyr	Val
1	217.2	98.4	54.0	19.8	24.1	13.3	40.4	52.1	367.3	622.8	NP	87.3	55.4	101.2	170.4	170.4	105.2	119.9	152.5	129.6	28.3	175.3	51.9	59.9	305.8
2	210.3	95.5	51.7	20.6	25.9	12.7	40.4	51.7	311.3	652.7	NP	88.1	52.0	91.3	165.0	177.9	98.4	109.7	147.9	130.9	26.7	178.7	50.8	58.9	304.7
3	223.6	104.2	47.2	21.8	25.9	13.9	42.4	54.7	380.9	689.8	NP	93.7	57.3	98.2	183.4	169.5	110.1	118.5	157.1	142.3	32.6	183.6	56.2	59.0	289.3
$\bar{x}$	217.0	99.4	50.9	20.7	25.3	13.3	41.1	52.8	353.2	655.1	NP	89.7	54.9	96.9	172.9	172.6	104.6	116.0	152.5	134.3	29.2	179.2	53.0	59.3	299.9
SD	6.7	4.5	3.5	1.0	1.1	0.6	1.2	1.6	36.9	33.5	NP	3.5	2.7	5.1	9.4	4.6	5.9	5.5	4.6	7.0	3.0	4.2	2.9	0.6	9.2
%CV	3.1	4.5	6.8	4.7	4.2	4.5	2.9	3.1	10.4	5.1	NP	3.9	4.9	5.3	5.5	2.7	5.6	4.8	3.0	5.2	10.4	2.3	5.4	0.9	3.1

NP = not performed. Specimens consisted of low and high control samples prepared in 0.1 M HCl, or a randomly selected patient plasma.

Table S6. Supplementary precision data – underivatized Isoleucine and *allo*-Ile.

	Interrun Low		Interrun High		Intrarun Low		Intrarun High	
#	allo	ile	allo	ile	allo	ile	allo	ile
1	27.5	24.0	263.5	118.3	27.0	25.7	263.5	118.3
2	27.0	25.7	266.8	122.5	27.1	26.0	285.8	121.2
3	22.5	27.5	264.1	118.2	25.6	24.6	276.4	118.1
$\bar{x}$	25.7	25.7	264.8	119.7	26.6	25.4	275.2	119.2
SD	2.8	1.7	1.7	2.5	0.9	0.7	11.2	1.7
%CV	**10.8**	**6.8**	**0.7**	**2.1**	**3.2**	**2.9**	**4.1**	**1.4**

Data based on low and high control samples prepared in 0.1 M HCl

Table S7. Method correlation – patient plasma (Page 1 of 4).

#	Ala			Arg			Asn			Cit			Gln		
	IEX/ UPLC (μM)	LCMS (μM)	Rec. (%)	IEX/ UPLC (μM)	LCMS (μM)	Rec. (%)	IEX/ UPLC (μM)	LCMS (μM)	Rec. (%)	IEX/ UPLC (μM)	LCMS (μM)	Rec. (%)	IEX/ UPLC (μM)	LCMS (μM)	Rec. (%)
1	133.4	140.1	105.0	77.5	85.3	110.1	26.1	33.7	128.9	24.8	26.7	107.6	389.7	395.9	101.6
2	106.8	106.2	99.5	109.6	110.4	100.7	55.4	65.6	118.4	41.2	39.5	96.0	419.9	407.3	97.0
3	203.1	195.9	96.5	152.3	153.9	101.1	54.9	64.9	118.3	44.3	43.2	97.5	373.5	373.9	100.1
4	348.0	363.2	104.4	96.2	96.0	99.8	31.9	44.1	138.2	30.9	31.7	102.7	512.9	536.6	104.6
5	457.4	476.9	104.3	140.4	150.0	106.9	24.3	29.3	120.5	23.3	23.3	100.0	634.8	662.8	104.4
6	344.4	383.4	111.3	98.8	110.8	112.2	25.5	35.4	138.8	20.9	23.2	110.9	561.3	601.1	107.1
7	226.2	220.2	97.3	50.5	53.0	105.0	35.1	35.7	101.8	14.6	17.2	117.6	443.1	450.4	101.7
8	240.9	239.2	99.3	132.2	128.9	97.5	54.9	57.6	105.0	46.7	50.0	107.2	890.5	999.2	112.2
9	474.8	488.4	102.8	130.3	128.0	98.3	54.4	59.4	109.1	17.7	23.6	132.9	652.5	689.0	105.6
10	329.6	306.2	92.9	98.4	90.5	91.9	29.6	32.8	110.6	21.3	26.5	124.5	364.2	406.4	111.6
11	334.3	348.3	104.2	93.0	87.5	94.1	43.4	51.6	118.8	10.4	11.1	106.7	679.5	726.7	107.0
12	476.1	421.2	88.5	83.3	87.6	105.1	30.8	40.0	129.6	26.2	30.5	116.3	605.7	572.6	94.5
13	387.7	375.4	96.8	133.7	145.5	108.9	31.2	35.2	112.8	23.8	25.9	108.8	529.0	490.3	92.7
14	159.7	159.7	100.0	65.8	70.6	107.3	46.9	50.8	108.5	13.5	16.1	119.4	660.0	789.0	119.5
15	394.8	394.3	99.9	112.7	108.5	96.3	32.5	38.4	118.0	17.7	21.0	118.9	593.0	598.7	101.0
16	456.0	445.6	97.7	72.4	76.4	105.5	48.9	50.5	103.3	25.2	23.0	91.5	680.3	575.9	84.7
17	507.9	532.1	104.8	132.4	131.2	99.1	57.0	60.6	106.5	28.4	30.2	106.5	647.4	545.7	84.3
18	380.8	378.8	99.5	74.5	75.0	100.7	50.8	51.9	102.2	15.1	15.5	102.9	644.2	582.5	90.4
19	275.9	294.4	106.7	100.2	105.1	104.9	93.9	70.6	75.2	15.7	16.0	102.3	566.8	518.8	91.5
20	239.3	208.0	86.9	95.2	106.7	112.1	16.0	15.3	96.2	12.2	12.3	100.8	419.8	442.9	105.5
21	446.8	406.8	91.0	139.5	134.4	96.4	67.1	68.8	102.4	20.5	18.4	89.6	793.4	778.1	98.1
22	251.8	206.7	82.1	142.0	124.3	87.5	21.9	19.9	90.9	10.9	9.4	86.7	401.6	394.4	98.2
23	320.4	273.8	85.5	100.4	144.9	144.3	26.6	24.3	91.2	10.6	8.6	81.3	547.3	565.1	103.3
24	377.1	339.7	90.1	205.9	194.5	94.5	21.5	18.8	87.4	13.1	11.7	88.8	410.5	403.5	98.3
25	169.5	150.7	88.9	117.1	86.5	73.9	15.9	13.1	82.5	5.4	5.0	93.8	360.1	357.6	99.3
26	139.9	107.5	76.8	61.2	69.8	114.1	12.9	9.5	73.5	6.7	6.7	100.8	246.2	271.3	110.2
27	189.6	172.6	91.0	34.0	60.9	179.3	16.7	14.8	88.4	3.6	3.2	87.3	305.5	364.8	119.4
28	341.8	326.4	95.5	60.5	73.4	121.5	24.9	23.5	94.5	13.7	13.9	101.7	609.8	696.0	114.1
29	293.3	273.7	93.3	81.4	80.7	99.1	45.7	47.4	103.7	11.0	9.1	82.5	439.2	490.9	111.8
30	214.9	217.2	101.1	94.6	98.4	104.0	20.6	19.8	96.5	12.0	13.3	110.5	520.4	622.8	119.7

Patient samples are numbered 1 to 30 (1-19 IEX tested samples, 20 to 30 UPLC tested samples).

Table S7. Method correlation – patient plasma (Page 2 of 4).

	Gly			His			Ile [a]			Leu			Lys		
#	IEX/ UPLC (μM)	LCMS (μM)	Rec. (%)	IEX/ UPLC (μM)	LCMS (μM)	Rec. (%)	IEX/ UPLC (μM)	LCMS (μM)	Rec. (%)	IEX/ UPLC (μM)	LCMS (μM)	Rec. (%)	IEX/ UPLC (μM)	LCMS (μM)	Rec. (%)
1	184.2	192.8	104.7	60.3	62.0	102.9	-	-	-	611.8	613.2	100.2	63.8	62.6	98.1
2	311.1	307.2	98.7	74.7	65.2	87.3	-	-	-	774.1	725.1	93.7	110.0	106.4	96.7
3	289.0	291.0	100.7	79.4	69.1	87.1	-	-	-	862.5	789.3	91.5	170.8	162.7	95.3
4	435.4	461.4	106.0	84.6	80.1	94.7	41.3	49.5	119.8	107.2	102.8	95.8	155.8	160.5	103.0
5	295.7	307.5	104.0	87.0	84.1	96.7	88.3	99.9	113.2	168.0	171.3	102.0	223.6	239.1	106.9
6	484.3	498.8	103.0	90.2	81.9	90.9	60.0	70.5	117.5	117.9	115.3	97.8	148.8	157.6	105.9
7	359.2	367.1	102.2	63.4	73.2	115.5	21.0	28.5	136.0	43.9	44.4	101.1	87.0	89.2	102.5
8	282.6	275.7	97.6	72.1	80.3	111.3	71.1	74.1	104.1	132.3	131.7	99.5	185.0	179.9	97.2
9	262.5	248.4	94.6	77.6	83.2	107.2	61.7	65.8	106.7	116.7	108.5	92.9	204.6	212.0	103.6
10	261.9	230.7	88.1	49.1	56.3	114.7	59.6	62.1	104.2	118.8	121.4	102.2	84.6	92.2	109.1
11	299.7	280.5	93.6	74.9	81.4	108.7	62.7	67.5	107.7	153.1	161.8	105.7	172.6	171.5	99.4
12	578.7	567.2	98.0	82.3	103.2	125.4	51.8	60.0	115.8	103.0	101.8	98.8	180.2	186.6	103.6
13	256.3	243.2	94.9	41.5	58.5	141.1	-	-	-	149.7	120.6	80.5	225.5	226.5	100.4
14	305.0	317.8	104.2	86.8	95.8	110.3	23.2	29.1	125.4	57.1	62.7	109.7	113.0	121.2	107.3
15	276.5	282.0	102.0	96.8	103.1	106.5	142.0	127.4	89.7	243.5	256.3	105.2	228.3	217.0	95.1
16	331.1	306.4	92.5	78.3	74.3	94.9	73.4	74.5	101.5	119.9	110.2	91.9	153.7	136.9	89.1
17	587.2	534.6	91.0	74.2	81.7	110.1	84.6	89.8	106.2	141.3	141.4	100.1	195.3	186.3	95.4
18	264.8	257.6	97.3	69.9	67.6	96.7	50.2	56.9	113.4	92.9	88.9	95.8	151.7	157.5	103.9
19	290.0	291.0	100.3	75.0	78.1	104.0	173.7	174.7	100.6	278.0	273.5	98.4	250.9	252.0	100.4
20	292.6	229.8	78.5	102.0	102.7	100.8	71.6	71.1	99.2	156.9	159.8	101.8	141.2	171.1	121.2
21	423.1	314.8	74.4	106.1	105.0	99.0	111.8	109.1	97.6	198.6	191.1	96.2	187.8	197.2	105.0
22	240.2	144.0	60.0	79.0	79.8	101.0	66.1	65.8	99.5	147.0	126.5	86.1	99.5	117.9	118.5
23	398.6	295.6	74.2	83.4	81.6	97.8	68.2	65.0	95.4	137.2	133.5	97.3	181.5	227.8	125.5
24	226.3	152.6	67.4	81.9	80.4	98.2	134.8	123.5	91.7	298.8	302.5	101.2	141.7	184.1	129.9
25	177.5	120.3	67.8	51.7	54.3	105.1	88.7	80.2	90.4	203.8	210.9	103.5	87.9	106.1	120.7
26	172.2	123.8	71.9	50.8	52.7	103.9	50.7	56.1	110.7	138.4	124.0	89.6	67.4	73.6	109.2
27	152.1	128.7	84.7	46.4	52.9	113.9	52.3	58.8	112.6	111.0	104.6	94.2	79.1	88.0	111.3
28	427.2	452.3	105.9	117.5	117.2	99.7	73.0	77.1	105.7	98.5	91.5	92.9	230.1	229.0	99.5
29	337.2	306.0	90.7	57.9	59.5	102.8	28.8	31.7	110.0	94.2	77.0	81.7	104.3	109.3	104.9
30	334.1	367.3	109.9	78.1	87.2	111.7	45.5	55.4	121.8	103.9	101.2	97.4	147.9	170.4	115.2

Patient samples are numbered 1 to 30 (1-19 IEX tested samples, 20 to 30 UPLC tested samples).

[a] Derivatized Ile analysis excluded in samples containing *allo*-Ile (as identified by IEX).

Table S7. Method correlation – patient plasma (Page 3 of 4).

#	Met			Orn			Phe			Pro			Ser		
	IEX/ UPLC (μM)	LCMS (μM)	Rec. (%)	IEX/ UPLC (μM)	LCMS (μM)	Rec. (%)	IEX/ UPLC (μM)	LCMS (μM)	Rec. (%)	IEX/ UPLC (μM)	LCMS (μM)	Rec. (%)	IEX/ UPLC (μM)	LCMS (μM)	Rec. (%)
1	9.3	9.1	98.1	48.3	51.0	105.6	51.0	51.4	100.8	112.6	136.9	121.5	117.7	123.6	105.0
2	21.4	19.8	92.6	84.9	83.5	98.4	67.4	69.5	103.1	127.9	144.9	113.3	179.0	178.4	99.7
3	29.2	30.1	103.1	108.4	95.6	88.2	107.3	104.0	96.9	306.2	385.1	125.8	178.9	185.0	103.5
4	30.5	25.0	81.8	52.9	61.9	117.0	49.2	52.9	107.6	255.5	277.6	108.7	179.8	186.5	103.8
5	22.8	20.5	90.0	104.3	114.0	109.3	47.4	52.3	110.3	248.7	264.9	106.5	191.2	198.6	103.9
6	19.8	19.3	97.4	66.9	74.5	111.4	220.5	251.5	114.0	118.3	144.8	122.4	164.7	178.3	108.3
7	15.7	14.6	93.0	90.6	95.1	105.0	51.5	52.7	102.5	76.7	91.4	119.2	267.6	269.8	100.8
8	26.7	25.8	96.6	59.2	57.6	97.3	70.7	62.5	88.3	312.0	300.7	96.4	178.6	163.8	91.7
9	32.9	28.5	86.7	60.6	60.6	100.1	69.0	63.8	92.5	89.3	86.2	96.5	173.6	148.2	85.4
10	19.2	14.0	73.2	47.5	43.9	92.4	73.0	61.0	83.5	175.6	170.5	97.1	172.4	146.3	84.9
11	24.3	19.6	80.5	97.2	102.4	105.3	58.4	53.3	91.2	164.6	183.7	111.6	143.6	134.3	93.5
12	31.6	28.5	90.3	78.3	80.9	103.4	38.1	37.2	97.7	159.1	167.9	105.5	194.1	200.1	103.1
13	30.5	28.8	94.4	109.7	118.7	108.2	215.5	204.8	95.0	141.1	156.4	110.8	150.9	154.2	102.2
14	13.9	21.2	152.4	83.7	89.7	107.2	67.1	66.3	98.7	181.7	178.3	98.1	129.1	130.9	101.4
15	27.6	25.7	93.2	95.8	110.7	115.6	404.2	406.8	100.7	246.1	254.8	103.5	201.0	181.8	90.5
16	24.3	22.3	91.6	71.3	65.2	91.4	60.7	58.1	95.8	71.5	73.1	102.2	117.7	111.0	94.3
17	41.8	37.1	88.7	82.3	79.8	97.0	96.3	94.3	97.9	289.3	306.3	105.9	245.9	242.1	98.5
18	18.7	20.9	111.9	69.4	67.6	97.4	47.6	42.5	89.4	737.1	761.0	103.2	181.0	180.2	99.6
19	45.5	44.7	98.1	67.2	61.3	91.2	83.0	88.2	106.3	153.7	172.3	112.1	196.7	200.2	101.8
20	32.2	28.9	89.8	132.6	97.7	73.6	129.4	118.1	91.2	73.7	82.8	112.4	142.0	116.9	82.3
21	57.4	56.7	98.8	106.1	96.7	91.1	88.4	76.1	86.1	63.9	75.7	118.4	236.7	197.2	83.3
22	28.1	27.7	98.3	42.6	33.7	79.0	138.4	124.3	89.8	140.5	136.5	97.1	158.4	124.2	78.4
23	48.9	46.9	96.0	206.0	127.0	61.7	125.5	106.8	85.1	172.7	189.4	109.7	167.2	133.3	79.7
24	32.0	30.0	93.6	82.5	77.9	94.5	141.9	132.0	93.1	86.6	106.2	122.7	159.7	133.2	83.4
25	14.2	14.0	98.4	124.3	118.6	95.5	102.5	92.1	89.9	118.4	152.5	128.9	93.0	69.7	75.0
26	8.0	8.3	103.2	56.0	41.9	74.7	73.4	64.2	87.5	234.8	256.9	109.4	83.6	53.4	63.9
27	16.5	18.3	111.0	158.2	162.6	102.8	202.0	196.7	97.4	252.6	228.0	90.3	109.9	83.5	76.0
28	72.8	74.4	102.2	180.0	190.4	105.8	126.7	120.7	95.3	341.3	342.9	100.5	218.6	191.5	87.6
29	18.3	19.8	107.8	65.9	65.4	99.2	99.7	77.4	77.7	229.6	245.3	106.9	187.0	158.5	84.8
30	27.5	31.0	112.8	84.6	105.2	124.4	113.8	119.9	105.4	272.7	261.8	96.0	135.1	129.6	95.9

Patient samples are numbered 1 to 30 (1-19 IEX tested samples, 20 to 30 UPLC tested samples).

Table S7. Method correlation – patient plasma (Page 4 of 4).

#	Tau			Thr			Trp			Tyr [b]			Val		
	IEX/ UPLC (μM)	LCMS (μM)	Rec. (%)	IEX/ UPLC (μM)	LCMS (μM)	Rec. (%)	IEX/ UPLC (μM)	LCMS (μM)	Rec. (%)	IEX/ UPLC (μM)	LCMS (μM)	Rec. (%)	IEX/ UPLC (μM)	LCMS (μM)	Rec. (%)
1	120.4	135.7	112.7	57.0	60.8	106.8	30.0	27.1	90.3	42.0	46.9	111.8	678.5	670.3	98.8
2	137.7	154.8	112.5	141.3	141.3	100.0	41.9	39.0	93.2	47.1	48.2	102.4	279.8	267.4	95.6
3	152.2	169.0	111.0	165.9	159.9	96.4	47.1	49.2	104.5	73.3	74.4	101.4	357.2	344.9	96.6
4	51.8	58.3	112.6	210.9	216.6	102.7	49.6	44.4	89.6	21.2	23.0	108.7	267.0	281.0	105.3
5	152.2	176.6	116.0	183.7	203.1	110.6	69.4	62.9	90.6	119.8	132.5	110.6	299.8	314.3	104.8
6	88.7	88.9	100.3	116.5	124.7	107.0	88.4	78.3	88.6	44.4	50.5	113.7	191.3	214.2	112.0
7	143.3	160.8	112.2	75.9	72.0	94.9	31.6	40.6	128.4	48.6	52.8	108.5	84.0	79.6	94.7
8	52.3	51.8	99.1	211.1	194.2	92.0	30.3	38.5	127.1	36.3	39.9	110.0	209.0	216.8	103.7
9	60.2	65.9	109.5	122.7	120.8	98.5	36.3	50.8	140.0	72.5	78.5	108.3	206.0	208.3	101.1
10	54.8	61.7	112.5	62.5	62.1	99.3	39.5	46.4	117.5	50.7	56.3	111.2	193.2	202.5	104.8
11	109.2	120.9	110.8	137.8	135.8	98.5	46.0	60.5	131.6	110.1	126.2	114.7	183.9	183.1	99.6
12	110.8	120.4	108.7	245.8	243.4	99.0	48.4	57.8	119.5	36.0	38.8	107.7	243.0	243.7	100.3
13	108.3	109.6	101.2	130.6	127.9	98.0	30.1	39.5	131.1	286.3	309.4	108.1	931.3	960.4	103.1
14	59.6	68.8	115.5	68.5	72.8	106.3	48.4	54.1	111.7	45.0	50.5	112.1	114.8	111.8	97.3
15	127.7	141.0	110.4	154.3	157.8	102.3	45.4	56.2	123.8	103.7	114.1	110.1	373.2	365.1	97.8
16	54.7	49.5	90.5	151.6	133.0	87.7	49.3	52.3	106.0	55.1	54.3	98.5	207.7	186.5	89.8
17	93.0	90.3	97.1	374.1	348.0	93.0	33.4	40.6	121.7	137.2	137.2	100.0	310.0	299.5	96.6
18	142.2	149.9	105.4	133.0	134.4	101.1	42.6	49.5	116.3	46.9	54.6	116.5	161.0	167.9	104.3
19	53.1	56.3	106.0	176.5	180.5	102.3	45.9	55.8	121.6	102.9	119.2	115.9	445.6	462.1	103.7
20	26.5	26.3	99.5	103.9	92.2	88.7	82.8	90.3	109.1	-	-	-	283.4	268.6	94.8
21	70.3	64.9	92.4	231.0	208.1	90.1	66.3	65.8	99.3	-	-	-	310.5	281.3	90.6
22	24.2	23.6	97.5	104.0	94.7	91.0	58.2	57.0	98.0	-	-	-	273.7	254.6	93.0
23	32.3	33.1	102.6	225.3	194.8	86.5	45.1	46.0	101.8	-	-	-	365.3	352.2	96.4
24	35.9	36.7	102.2	95.6	82.3	86.1	73.6	76.6	104.0	-	-	-	484.6	453.0	93.5
25	25.4	25.3	99.5	84.0	70.0	83.4	64.1	67.2	104.9	-	-	-	362.4	337.9	93.3
26	41.8	46.3	110.8	39.8	36.4	91.3	34.1	39.6	116.1	29.2	37.2	127.4	296.1	294.7	99.5
27	22.0	21.9	99.5	60.7	65.8	108.3	70.8	78.1	110.4	38.2	47.0	123.0	271.5	307.0	113.1
28	43.3	45.7	105.4	193.3	199.1	103.0	43.4	46.3	106.7	95.6	109.3	114.4	254.2	268.1	105.4
29	27.4	27.6	100.8	110.3	111.1	100.7	45.4	51.9	114.3	58.4	67.2	115.0	150.7	145.8	96.8
30	24.8	28.3	114.5	163.8	175.3	107.0	45.4	50.8	111.8	42.3	59.9	141.6	264.7	305.8	115.5

Patient samples are numbered 1 to 30 (1-19 IEX tested samples, 20 to 30 UPLC tested samples).
[b] Tyr was not reported for a subset of UPLC results due to limitations of the method.

Table S8. Ion Suppression/ Matrix Effect Data Analysis [a]

S#	Ala	Arg	Asp	Cys	Glu	Gly	His	Ile	Leu	Lys	Met	Phe	Pro	Ser	Thr	Tyr	Val
1	29.2	27.2	27.5	38.9	24.3	24.4	26.4	24.7	24.7	24.6	25.0	23.1	38.9	21.8	23.2	29.0	24.1
2	23.5	25.9	26.3	33.8	23.7	24.5	30.0	20.9	22.4	25.2	26.4	26.6	33.8	21.1	21.9	26.4	24.8
3	30.6	28.2	28.2	39.0	26.4	30.1	27.3	25.4	24.1	28.0	31.5	27.9	39.0	27.4	23.7	28.5	30.1
4	53.5	49.4	53.2	56.1	52.2	49.8	55.0	55.0	53.7	54.5	52.3	54.0	56.3	48.3	41.2	53.7	51.2
5	43.0	38.3	43.1	39.6	40.8	37.5	44.6	44.0	42.0	43.2	40.7	46.1	49.4	36.9	28.6	41.2	41.1
6	34.7	25.9	35.1	35.5	32.4	30.3	32.3	36.1	32.3	34.9	30.4	34.4	40.2	23.0	17.6	33.1	35.6
7	26.1	21.1	29.8	23.3	78.1	26.8	25.4	28.3	24.2	27.7	30.3	28.8	35.4	27.0	20.1	24.3	33.2
8	7.8	17.4	12.1	16.8	5.4	12.8	16.1	10.3	7.9	13.8	19.5	15.5	18.8	19.4	11.3	15.7	9.1
9	27.8	27.9	27.1	31.2	29.3	27.4	29.0	28.3	25.7	29.7	31.1	29.0	39.3	25.4	27.6	29.3	29.2
10	33.0	33.7	35.3	36.2	35.9	36.6	36.4	34.6	35.9	35.5	38.3	38.3	40.5	40.5	33.3	35.3	32.2
11	23.2	24.5	24.0	26.4	26.1	29.1	25.2	25.3	22.6	28.0	27.8	26.3	33.7	29.0	20.0	26.8	26.0
12	27.6	25.2	27.7	30.1	26.0	28.7	27.9	26.7	24.1	28.3	29.5	32.6	34.6	28.6	25.8	27.5	25.3
13	30.7	29.3	28.6	30.6	22.3	27.1	31.7	26.2	29.6	30.2	28.5	31.8	38.2	37.8	29.3	27.8	21.4
14	16.1	18.2	18.3	21.1	17.6	17.8	16.3	14.8	11.2	18.4	20.7	17.7	26.1	20.0	16.4	19.7	17.1
15	27.6	23.3	26.3	25.7	21.5	23.6	26.2	23.0	20.7	26.3	26.4	29.3	31.5	32.0	25.4	29.3	24.1
16	25.6	20.0	24.8	27.0	24.0	26.7	26.6	17.5	20.8	26.8	28.3	26.9	33.8	37.6	23.6	29.2	21.3
17	16.6	18.4	24.6	23.2	20.0	23.6	19.3	24.6	22.7	29.6	22.0	25.8	34.0	26.5	24.6	22.3	23.1
18	6.5	6.1	9.4	13.2	8.4	4.1	8.4	13.7	13.0	13.9	11.5	45.9	15.5	13.2	9.5	9.0	10.1
19	23.2	22.9	26.9	24.6	20.9	26.3	24.5	23.0	24.0	31.3	28.7	25.8	40.4	45.4	22.7	25.7	27.3
20	45.5	41.2	40.4	44.3	43.2	44.8	42.4	47.6	42.3	47.3	46.7	45.9	51.9	62.5	40.6	46.1	42.7
21	21.4	21.6	20.1	19.9	18.7	21.4	21.7	20.2	21.9	26.6	24.3	22.2	32.5	54.0	21.0	22.6	18.3
22	25.4	31.3	29.2	31.5	28.7	35.2	33.1	32.1	30.9	31.2	30.0	31.4	34.8	28.2	30.2	30.2	28.8
23	22.0	24.6	23.1	21.1	19.3	23.2	20.2	24.9	21.7	27.4	23.3	26.1	34.4	54.0	17.8	25.0	24.1
24	24.9	26.7	26.9	27.2	23.6	22.4	25.0	23.9	26.8	31.9	30.1	27.7	41.3	54.5	23.7	25.8	26.3
25	19.3	18.7	21.4	19.2	17.5	21.2	19.1	21.2	18.0	23.6	21.5	23.7	35.4	51.4	15.0	24.3	20.6
26	21.7	15.2	27.9	21.3	18.8	22.8	17.9	14.0	19.5	21.3	24.9	22.7	32.7	22.3	20.0	21.9	19.6
27	29.8	22.7	30.3	28.2	30.8	22.7	27.8	30.2	26.9	31.9	25.0	30.1	33.9	20.6	26.8	27.5	23.1
28	25.7	23.3	25.8	25.9	24.9	17.4	25.1	27.1	25.7	26.6	23.7	25.8	33.9	20.0	22.2	24.9	20.7
29	23.7	31.8	24.5	25.8	31.4	21.3	28.1	30.8	30.8	33.6	32.7	28.3	32.5	30.3	26.8	28.9	29.4
30	29.2	29.8	25.0	29.3	26.2	21.6	22.7	24.7	30.6	30.8	30.2	31.2	34.5	31.5	24.8	28.4	27.3
Min	6.5	6.1	9.4	13.2	5.4	4.1	8.4	10.3	7.9	13.8	11.5	15.5	15.5	13.2	9.5	9.0	9.1
Max	53.5	49.4	53.2	56.1	78.1	49.8	55.0	55.0	53.7	54.5	52.3	54.0	56.3	62.5	41.2	53.7	51.2
Avg	26.5	25.7	27.4	28.9	27.3	26.0	27.1	26.6	25.9	29.4	28.7	30.0	35.9	33.0	23.8	28.0	26.2

[a] Displayed value = (IS Area of STD - IS Area of Plasma Sample)/(IS Area of STD in 0.1 M HCl). The values account for all matrix effects in sample preparation plus ion suppression for the C13,15 labeled internal standard compounds (Cambridge Isotope MSK-A2).

Table S9. Spiked plasma analysis

AA [b]	Level 1 [c]			Level 2			Level 3			Level 4			Level 5			Level 6		
	Exp. (µM)	Lcms (µM)	Rec. (%)	Exp. (µM)	Lcms (µM)	Rec. (%)	Exp. (µM)	Lcms (µM)	Rec. (%)	Exp. (µM)	Lcms (µM)	Rec. (%)	Exp. (µM)	Lcms (µM)	Rec. (%)	Exp. (µM)	Lcms (µM)	Rec. (%)
Ala	265.0	272.6	102.9	290.0	288.4	99.5	322.0	322.2	100.1	359.5	378.9	105.4	515.0	507.3	98.5	697.0	709.0	101.7
Arg	127.5	132.5	103.9	132.5	137.7	103.9	172.5	184.0	106.7	481.2	459.7	95.5	1312.5	1255.6	95.7	N/A	N/A	N/A
ASA	25.0	28.4	113.5	50.0	54.0	107.9	166.7	165.4	99.2	200.0	210.1	105.1	322.6	355.7	110.3	500.0	568.8	113.8
Asn	44.1	47.9	108.5	63.5	69.8	109.9	81.6	76.6	93.8	88.5	69.7	78.7	313.5	231.1	73.7	419.1	385.7	92.0
Asp	11.2	15.3	136.6	14.1	15.7	111.1	39.1	37.2	95.0	48.7	49.9	102.4	264.1	268.3	101.6	386.2	404.1	104.6
Cit	13.2	12.7	96.5	14.4	13.9	96.5	39.4	33.0	83.8	50.7	45.5	89.8	264.4	225.6	85.3	388.2	336.7	86.7
Cys	17.8	21.5	120.8	27.3	27.5	100.8	36.5	32.8	89.9	39.8	41.3	103.7	152.3	148.3	97.4	205.3	209.0	101.8
Glu	36.1	37.7	104.5	57.9	64.5	111.4	61.1	59.8	98.0	95.4	91.9	96.3	286.1	266.6	93.2	432.9	438.7	101.3
Gly	219.0	218.2	99.6	256.5	241.6	94.2	261.9	263.9	100.8	286.9	289.7	101.0	511.9	490.9	95.9	594.0	560.4	94.4
Gln	561.0	559.7	99.8	669.5	675.3	100.9	723.4	679.4	93.9	748.6	658.3	87.9	875.9	799.2	91.2	1231.3	1098.8	89.2
HCY	5.0	5.0	99.4	10.0	8.1	81.0	50.0	46.5	93.0	375.0	374.0	99.7	1250.0	1230.5	98.4	N/A	N/A	N/A
His	105.3	107.2	101.8	110.3	126.0	114.2	150.3	165.2	109.9	462.0	484.8	104.9	1301.2	1310.2	100.7	N/A	N/A	N/A
HCT	11.4	11.4	99.5	28.6	27.8	97.4	57.1	53.4	93.4	142.9	159.4	111.6	428.6	434.8	101.5	857.1	876.8	102.3
Ile	42.7	46.3	108.6	80.2	84.0	104.8	156.4	157.5	100.7	181.4	187.1	103.2	406.4	420.5	103.5	417.7	440.6	105.5
Leu	75.6	77.1	102.0	113.1	107.9	95.4	232.5	239.1	102.8	270.0	282.7	104.7	450.6	421.6	93.6	607.5	507.6	83.6
Lys	214.6	212.3	98.9	219.6	226.3	103.1	259.6	255.1	98.3	556.8	550.2	98.8	1356.9	1327.2	97.8	N/A	N/A	N/A
Met	17.8	17.7	99.7	40.2	40.3	100.2	55.3	52.5	95.0	65.2	62.0	95.2	290.2	252.4	87.0	392.8	356.5	90.8
Orn	157.3	172.5	109.6	162.3	165.5	102.0	202.3	207.8	102.7	507.1	514.1	101.4	1327.7	1323.8	99.7	N/A	N/A	N/A
Pro	208.5	229.5	110.1	235.6	249.7	106.0	246.0	255.7	103.9	260.6	269.1	103.3	485.6	470.0	96.8	583.5	602.3	103.2
Phe	36.2	36.0	99.5	73.7	66.9	90.8	79.4	81.1	102.2	104.4	97.4	93.3	329.4	311.7	94.6	411.2	362.5	88.2
Ser	153.0	170.5	111.4	180.2	197.2	109.4	190.5	202.9	106.5	205.2	210.6	102.7	430.2	427.7	99.4	528.0	556.8	105.5
Tau	50.7	47.1	92.9	75.7	67.9	89.8	127.4	129.5	101.6	164.9	156.2	94.7	300.7	264.2	87.9	502.4	507.8	101.1
Thr	114.3	118.1	103.3	151.8	148.3	97.7	162.5	157.0	96.6	187.5	184.7	98.5	412.5	390.1	94.6	489.3	459.2	93.9
Trp	58.7	56.8	96.9	63.7	60.8	95.5	103.7	96.3	92.9	421.6	359.5	85.3	1277.4	1074.8	84.1	N/A	N/A	N/A
Tyr	46.4	45.0	96.9	83.9	72.5	86.4	107.3	101.2	94.3	132.3	128.5	97.1	357.3	343.9	96.3	421.4	404.9	96.1
Val	142.7	143.9	100.9	180.2	183.1	101.6	415.9	427.7	102.8	440.9	420.7	95.4	517.7	527.5	101.9	665.9	661.2	99.3

For Ala, Asn, Asp, Cit, Cys, Glu, Gly, Ile, Leu, Met, Phe, Pro, Ser, Tau, Thr, Tyr, and Val, %plasma (v/v) equaled either 90% or 85% across all levels. For Arg, His, homocystine (HCY), Lys, Orn, and Trp, %plasma = 96%, 96%, 96%, 85%, and 50% for levels 1 to 5, respectively. For argininosuccinic acid (ASA) and Gln %plasma ranged from 86 to 98%. Homocitrulline spiked plasmas were >91% plasma. For all amino acids except Gln, expected concentration (Exp.) was determined by extrapolation based on LCMS measurement of the unspiked plasma. For Gln expected concentration equaled ion-exchange chromatography measurement for that sample (LCMS measurement of unspiked plasma was not performed). Additional high levels were tested for ASA – 1142.9 µM (Rec. = 101.3%,) and 1428.57 µM (rec. = 107.4%), as well as for Gln – 1701.0 µM (Rec. =92.8%) and 1771.5 µM (Rec. = 99.2%).

Table S10. Spiked urine analysis

AA	Level 1			Level 2			Level 3			Level 4			Level 5			Level 6			Level7		
	Exp. (µM)	Lcms (µM)	Rec. (%)	Exp. (µM)	Lcms (µM)	Rec. (%)	Exp. (µM)	Lcms (µM)	Rec. (%)	Exp. (µM)	Lcms (µM)	Rec. (%)	Exp. (µM)	Lcms (µM)	Rec. (%)	Exp. (µM)	Lcms (µM)	Rec. (%)	Exp. (µM)	Lcms (µM)	Rec. (%)
**Arg**	23	22.1	96.2	34.5	30.2	87.6	95.1	91.6	96.4	265	282.4	106.5	291.4	295.3	101.3	513.4	487.3	94.9	1258.4	1245.5	99
**Lys**	51.5	50.7	98.5	77.3	76.1	98.5	151	141.1	93.5	287.2	268.2	93.4	342.7	341.5	99.6	533.1	524.1	98.3	1270.7	1167.1	91.9
**Orn**	3.7	4	108.8	5.5	4.6	83.4	57.2	56.1	98.1	255.2	265.2	103.9	256.6	259.5	101.1	504.6	554.7	109.9	1252.9	1453.7	116

Expected concentrations were extrapolated from LCMS result for unspiked/undiluted specimen. Spiked urine specimens (Levels 1-7) consisted of 50, 75, 98, 90, 90, 80, and 50% urine.

Table S11. Spiked CSF analysis

AA	Level 1			Level 2			Level 3			Level 4			Level 5			Level 6		
	Exp. (µM)	Lcms (µM)	Rec. (%)	Exp. (µM)	Lcms (µM)	Rec. (%)	Exp. (µM)	Lcms (µM)	Rec. (%)	Exp. (µM)	Lcms (µM)	Rec. (%)	Exp. (µM)	Lcms (µM)	Rec. (%)	Exp. (µM)	Lcms (µM)	Rec. (%)
**Gly**	4.1	4.4	107.2	6.2	6.4	104.2	11.7	12.4	105.8	58.1	58.1	100.1	257.4	262	101.8	264	250.3	94.8
**Ser**	17.5	17.5	99.9	26.3	26.3	100.1	28.2	34.4	121.8	84.4	84.6	100.3	281.6	283.8	100.8	279.8	258.2	92.3

Expected concentrations were extrapolated from LCMS result for unspiked/undiluted specimen. Spiked CSF specimens (Levels 1-6) consisted of 50, 75, 75, 98, 90, and 90% CSF

Table S12. Unlabeled Isoleucine and *allo*-Ile in plasma analysis.

	Target Concentrations		Result Concentrations		Recovery (%)	
Sample	$[ILE]_{Target}$	$[allo]_{Target}$	$[Ile]_{Result}$	$[allo]_{Result}$	Ile	allo
1	1044	1000	978.4	985.88	**93.9**	**98.59**
2	44	66.7	45.27	71.25	**107.41**	**106.82**
3	403.02	400.84	407.29	395.17	**101.06**	**98.59**
4	927.25	300.63	814.77	293.1	**87.87**	**97.49**
5	1451	200.4	1341.41	199.83	**92.45**	**99.71**
6	82.85	7.35	84.73	8.82	**102.27**	**119.99**
7	129.07	16.22	124.2	14.07	**96.22**	**86.74**
8	88.1	7.14	89.9	7.5	**102.05**	**104.98**
9	130.73	9.2	120.53	9.03	**92.19**	**98.11**

Samples 3 and 6-9 (100% plasma) results based on IEX analysis of patient plasma (Maple Syrup Urine Disease patient plasmas). Additional samples 1, 2, 4, and 5 concentrations extrapolated based on sample spiking with standard or water (50, 99, 75, 50% plasma, respectively).

Table S13. Matrix-specific precision and signal suppression results (Page 1 of 5).

TESTING IN PLASMA

ALANINE IN PLASMA (IEX REFERENCE VALUE = 421.4 uM)

Run	Replicate A	Replicate B	Replicate C	Avg	STDdev	%CV (Intrarun)
1	450.7	464.9	459.7	458.4	7.2	1.6
2	454.9	460.8	457.3	457.7	2.9	0.6
3	439.0	414.2	425.0	426.1	12.4	2.9
4	434.4	419.8	454.4	436.2	17.4	4.0
5	434.38	419.76	454.42	436.2	17.4	4.0

n = 15 replicates	Avg	STDdev	%CV
	**442.9**	**17.2**	**3.9**

I.S. ALANINE C13, N15 (MSK-A2)

STD I.S. INTENSITY	REPLICATE A I.S. INTENSITY	REPLICATE B I.S. INTENSITY	REPLICATE C I.S. INTENSITY	SUPPR. A	SUPPR. B	SUPPR. C	*SUPPR. Avg*
4239283	3033192.7	3103082.6	2911927.9	28.5	26.8	31.3	28.9
896780.7	1921769.9	2132271.3	2070579.1	-114.3	-137.8	-130.9	-127.7
4670792	2591238.1	3183753.4	3569416.7	44.5	31.8	23.6	33.3
4981035.2	2695016.6	2759158	3045964.7	45.9	44.6	38.8	43.1
5659569.4	3290917.6	2781605.4	3726108.2	41.9	50.9	34.2	42.3

ARGININE IN PLASMA (IEX REFERENCE VALUE = 124.3 uM)

Run	Replicate A	Replicate B	Replicate C	Avg	STDdev	%CV (Intrarun)
1	116.18	130.03	126.33	124.2	7.2	5.8
2	129.51	126.54	129.79	128.6	1.8	1.4
3	129.2	129.59	127.46	128.8	1.1	0.9
4	126.77	120.95	130.8	126.2	5.0	3.9
5	126.96	124.69	133.38	128.3	4.5	3.5

n = 15 replicates	Avg	STDdev	%CV
	**127.2**	**4.2**	**3.3**

I.S. ARGININE C13, N15 (MSK-A2)

STD I.S. INTENSITY	REPLICATE A I.S. INTENSITY	REPLICATE B I.S. INTENSITY	REPLICATE C I.S. INTENSITY	SUPPR. A	SUPPR. B	SUPPR. C	*SUPPR. Avg*
2548978.6	2134102	2216475.8	2027676.3	16.3	13.0	20.5	16.6
664968.5	1560783.8	1712207.4	1561123.3	-134.7	-157.5	-134.8	-142.3
1834163.4	1174189.2	1484358.7	1786092.7	36.0	19.1	2.6	19.2
2476249.2	1465223.9	1653480.4	1711569.3	40.8	33.2	30.9	35.0
2344328.8	1685389.3	1524421.1	2329400.3	28.1	35.0	0.6	21.2

ASPARAGINE IN PLASMA (IEX REFERENCE VALUE = 45.4 uM)

Run	Replicate A	Replicate B	Replicate C	Avg	STDdev	%CV (Intrarun)
1	42.2	35.7	44.6	40.9	4.6	11.2
2	46.6	48.2	49.0	47.9	1.2	2.6
3	49.8	42.5	39.0	43.8	5.5	12.5
4	43.2	48.4	47.4	46.3	2.8	6.0
5	45.8	43.8	50.0	46.5	3.2	6.9

n = 15 replicates	Avg	STDdev	%CV
	**45.1**	**4.1**	**9.1**

ASPARTIC ACID IN PLASMA (IEX REFERENCE VALUE = 25.0 uM)

Run	Replicate A	Replicate B	Replicate C	Avg	STDdev	%CV (Intrarun)
1	23.0	22.8	24.4	23.4	0.8	3.6
2	25.4	24.5	25.7	25.2	0.6	2.4
3	18.0	26.2	24.7	23.0	4.3	18.9
4	25.5	27.2	29.1	27.3	1.8	6.5
5	24.1	24.8	23.4	24.1	0.7	2.8

n = 15 replicates	Avg	STDdev	%CV
	**24.6**	**2.4**	**9.9**

I.S. ASPARTIC ACID C13, N15 (MSK-A2)

STD I.S. INTENSITY	REPLICATE A I.S. INTENSITY	REPLICATE B I.S. INTENSITY	REPLICATE C I.S. INTENSITY	SUPPR. A	SUPPR. B	SUPPR. C	*SUPPR. Avg*
2336347	1907309.4	1919787.8	1828197.4	18.4	17.8	21.7	19.3
553561	1227945.6	1353338.4	1278198.9	-121.8	-144.5	-130.9	-132.4
18835.2	13289.6	11563.3	13805.4	29.4	38.6	26.7	31.6
2631483	1632038.7	1755407.3	1794657.1	38.0	33.3	31.8	34.4
2618287.5	1797291.8	1566619	2301878.4	31.4	40.2	12.1	27.9

CITRULLINE IN PLASMA (IEX REFERENCE VALUE = 25.3 uM)

Run	Replicate A	Replicate B	Replicate C	Avg	STDdev	%CV (Intrarun)
1	27.8	26.12	24.94	26.3	1.4	5.5
2	25.17	24.6	24.65	24.8	0.3	1.3
3	25.19	**31.18**	28.65	28.3	3.0	10.6
4	**26.46**	26.84	27.29	26.9	0.4	1.5
5	**24.97**	24.2	21.53	23.6	1.8	7.7

n = 15 replicates	Avg	STDdev	%CV
	**26.0**	**2.2**	**8.6**

CYSTINE IN PLASMA (IEX REFERENCE VALUE = 8.9 uM)

Run	Replicate A	Replicate B	Replicate C	Avg	STDdev	%CV (Intrarun)
1	11.2	10.9	10.2	10.8	0.5	5.1
2	12.1	11.8	14.4	12.8	1.4	11.1
3	12.1	11.5	13.8	12.5	1.2	9.3
4	12.4	13.8	13.6	13.2	0.8	5.9
5	9.9	11.7	10.8	10.8	0.9	8.3

n = 15 replicates	Avg	STDdev	%CV
	**12.0**	**1.4**	**11.3**

I.S. CYSTINE C13, N15 (MSK-A2)

STD I.S. INTENSITY	REPLICATE A I.S. INTENSITY	REPLICATE B I.S. INTENSITY	REPLICATE C I.S. INTENSITY	SUPPR. A	SUPPR. B	SUPPR. C	*SUPPR. Avg*
177806.7	142065.4	144687.2	141269.2	20.1	18.6	20.5	19.8
54813.9	119102.4	127517.6	122592.4	-117.3	-132.6	-123.7	-124.5
157705.7	88244.1	111932.7	126222.5	44.0	29.0	20.0	31.0
221130.7	129885.4	139678.9	152663.6	41.3	36.8	31.0	36.4
223961.5	143502.4	133119.3	187612	35.9	40.6	16.2	30.9

GLUTAMIC ACID IN PLASMA (IEX REFERENCE VALUE = 76.2 uM)

Run	Replicate A	Replicate B	Replicate C	Avg	STDdev	%CV (Intrarun)
1	74.6	72.2	71.9	72.9	1.5	2.0
2	78.9	79.1	79.9	79.3	0.5	0.6
3	74.2	72.9	74.3	73.8	0.8	1.1
4	85.4	91.7	94.2	90.4	4.5	5.0
5	80.6	85.9	81.8	82.8	2.7	3.3

n = 15 replicates	Avg	STDdev	%CV
	**79.8**	**7.0**	**8.7**

I.S. GLUTAMIC ACID C13, N15 (MSK-A2)

STD I.S. INTENSITY	REPLICATE A I.S. INTENSITY	REPLICATE B I.S. INTENSITY	REPLICATE C I.S. INTENSITY	SUPPR. A	SUPPR. B	SUPPR. C	*SUPPR. Avg*
2270872.5	***1813055.5***	1867692.6	1785077.5	20.2	17.8	21.4	19.8
596263.3	1216254.6	1341932.1	***1256230.8***	-104.0	-125.1	-110.7	-113.2
2201997.7	1342818.1	1618271.5	1883198.1	39.0	26.5	14.5	26.7
2809685.3	1443600.2	1574784.2	1582395.8	48.6	44.0	43.7	45.4
2421782.2	1775977.8	1548322.8	2282776.9	26.7	36.1	5.7	22.8

Samples consist of pooled plasma, urine, or CSF, constituting equal volume mixtures from 5-6 separate patients. Samples were analyzed in triplicate and run in 5 separate batches over the course of approximately 1 week, using fresh aliquots of plasma, urine, and CSF stored at -80°C for each run. The IEX reference value reflects single analysis of an aliquot of pooled sample using ion-exchange chromatography (Biochrom Analyzer). Differences in results between the platforms will depend on matrix-specific features and day-to-day performance, including but not limited to quality of analyte separation during IEX analysis and underlying interferents in the pooled samples affecting optical-based detection. Data for aspartic acid and proline are shown for urine due to relevance of the C13,N15 internal standards of these targets to ornithine (Pro closest in retention time, Asp appeared most similar overall to Orn as internal control during initial testing). In the matrix suppresion analysis, suppression = (STD I.S. Intensity – Replicate I.S. Intensity) / (STD I.S. Intensity). Run 2 was a single outlier for which suppression of internal standard signal in 0.1 M HCl compared to matrix was not observed. For the remaining runs, suppression was generally greater in the pooled plasma sample compared to urine and CSF. Sample preparation error (*) resulted in no chromatography peaks in one CSF sample analysis (Run 2, Replicate C).

Table S13. Matrix-specific precision and signal suppression results (Page 2 of 5).

TESTING IN PLASMA (CONTINUED)

GLUTAMINE IN PLASMA (IEX REFERENCE VALUE = 517.5 uM)

Run	Replicate A	Replicate B	Replicate C	Avg	STDdev	%CV (Intrarun)
1	510.2	486.0	470.5	488.9	20.0	4.1
2	551.0	525.7	536.6	537.7	12.7	2.4
3	469.6	528.3	532.7	510.2	35.2	6.9
4	507.5	527.1	541.5	525.3	17.1	3.2
5	488.8	488.8	519.0	498.9	17.5	3.5

n = 15 replicates	Avg	STDdev	%CV
	**512.2**	**26.0**	**5.1**

GLYCINE IN PLASMA (IEX REFERENCE VALUE = 375.5 uM)

Run	Replicate A	Replicate B	Replicate C	Avg	STDdev	%CV (Intrarun)
1	399.1	364.5	365.7	376.4	19.6	5.2
2	400.9	420.6	417.7	413.1	10.6	2.6
3	339.5	406.3	380.3	375.3	33.7	9.0
4	420.2	432.4	431.6	428.1	6.8	1.6
5	401.3	396.3	400.2	399.2	2.6	0.7

n = 15 replicates	Avg	STDdev	%CV
	**398.4**	**26.3**	**6.6**

I.S. GLYCINE C13, N15 (MSK-A2)

STD I.S. INTENSITY	REPLICATE A I.S. INTENSITY	REPLICATE B I.S. INTENSITY	REPLICATE C I.S. INTENSITY	SUPPR. A	SUPPR. B	SUPPR. C	*SUPPR. Avg*
3637497.2	2635663.7	2898375.6	2893975	27.5	20.3	20.4	22.8
820876.3	1856534.7	2018806.5	1903319.8	-126.2	-145.9	-131.9	-134.7
4370385.6	2336059.5	2607505.7	3101378.7	46.5	40.3	29.0	38.6
4060489.5	2508443.7	2724014.5	2862251	38.2	32.9	29.5	33.5
4053265.6	2951107.4	2576231.9	3671544.3	27.2	36.4	9.4	24.4

HISTADINE (IEX REFERENCE VALUE = 90.6 uM)

Run	Replicate A	Replicate B	Replicate C	Avg	STDdev	%CV (Intrarun)
1	95.0	100.2	102.3	99.2	3.8	3.8
2	99.3	100.7	99.9	100.0	0.7	0.7
3	97.0	98.8	97.0	97.6	1.0	1.1
4	94.0	98.4	96.9	96.4	2.3	2.3
5	96.3	91.5	93.7	93.8	2.4	2.6

n = 15 replicates	Avg	STDdev	%CV
	**97.4**	**3.0**	**3.1**

I.S. HISTADINE C13, N15 (MSK-A2)

STD I.S. INTENSITY	REPLICATE A I.S. INTENSITY	REPLICATE B I.S. INTENSITY	REPLICATE C I.S. INTENSITY	SUPPR. A	SUPPR. B	SUPPR. C	*SUPPR. Avg*
2515249.9	1949196	1957211.7	1888558.2	22.5	22.2	24.9	23.2
476136.5	1170742.9	1261126.9	1128813.5	-145.9	-164.9	-137.1	-149.3
1862665.2	1286398.8	1596791.6	1765297.2	30.9	14.3	5.2	16.8
2217598.1	1515241.6	1692087.7	1796732.6	31.7	23.7	19.0	24.8
2706661.2	1788217.9	1682682.5	2318228.3	33.9	37.8	14.4	28.7

ISOLEUCINE (UNDERIVATIZED) IN PLASMA (IEX REFERENCE VALUE = 123.2 uM)

Run	Replicate A	Replicate B	Replicate C	Avg	STDdev	%CV (Intrarun)
1	145.7	105.1	151.1	133.9	25.2	18.8
2	140.3	82.9	110.1	111.1	28.7	25.9
3	169.8	130.4	124.5	141.6	24.6	17.4
4	116.2	132.4	128.9	125.8	8.6	6.8
5	153.6	149.2	132.6	145.1	11.0	7.6

n = 15 replicates	Avg	STDdev	%CV
	**131.5**	**22.0**	**16.7**

I.S. ISOLEUCINE C13, N15 (MSK-A2)

STD I.S. INTENSITY	REPLICATE A I.S. INTENSITY	REPLICATE B I.S. INTENSITY	REPLICATE C I.S. INTENSITY	SUPPR. A	SUPPR. B	SUPPR. C	*SUPPR. Avg*
6713.5	4716.5	6980.9	4421.3	29.7	-4.0	34.1	20.0
1371.2	2492.7	4847.3	3676.3	-81.8	-253.5	-168.1	-167.8
5301.1	***4598.2***	3402.7	5103	13.3	35.8	3.7	17.6
8387	7585.4	6035	7531.5	9.6	28.0	10.2	15.9
8839.1	5720.6	5163.7	7946.2	35.3	41.6	10.1	29.0

ALLO-ISOLEUCINE I N PLASMA (IEX REFERENCE VALUE = 45.2 uM)

Run	Replicate A	Replicate B	Replicate C	Avg	STDdev	%CV (Intrarun)
1	49.7	33.7	49.9	44.4	9.3	20.9
2	48.2	29.1	36.8	38.0	9.6	25.3
3	59.0	46.5	43.1	49.5	8.4	16.9
4	40.8	48.6	44.3	44.5	3.9	8.7
5	57.6	55.5	48.7	53.9	4.7	8.6

n = 15 replicates	Avg	STDdev	%CV
	**46.1**	**8.5**	**18.4**

LEUCINE IN PLASMA (IEX REFERENCE VALUE = 284.8 uM)

Run	Replicate A	Replicate B	Replicate C	Avg	STDdev	%CV (Intrarun)
1	235.7	247.0	252.7	245.1	8.7	3.5
2	288.0	256.7	293.7	279.5	19.9	7.1
3	290.3	300.2	287.7	292.7	6.6	2.2
4	295.0	281.7	293.2	290.0	7.2	2.5
5	269.6	278.3	288.7	278.8	9.6	3.4

n = 15 replicates	Avg	STDdev	%CV
	**277.2**	**20.1**	**7.2**

I.S. LEUCINE C13, N15 (MSK-A2)

STD I.S. INTENSITY	REPLICATE A I.S. INTENSITY	REPLICATE B I.S. INTENSITY	REPLICATE C I.S. INTENSITY	SUPPR. A	SUPPR. B	SUPPR. C	*SUPPR. Avg*
4967912.3	3598454	3943537.7	3556110.6	27.6	20.6	28.4	25.5
1154246.5	2464657.7	2834228.9	2451120.3	-113.5	-145.5	-112.4	-123.8
3103563.3	1696966.6	2102830.5	2347898.5	45.3	32.2	24.3	34.0
5050446.2	3221690.4	3669959.8	3850582	36.2	27.3	23.8	29.1
5171612.6	4007319.4	3488427.8	4653901.6	22.5	32.5	10.0	21.7

LYSINE IN PLASMA (IEX REFERENCE VALUE = 195.5 uM)

Run	Replicate A	Replicate B	Replicate C	Avg	STDdev	%CV (Intrarun)
1	187.3	204.7	193.2	195.0	8.9	4.5
2	200.4	196.9	207.3	201.5	5.3	2.6
3	190.4	196.9	193.2	193.5	3.3	1.7
4	203.6	208.0	210.2	207.3	3.4	1.6
5	195.2	202.6	204.6	200.8	4.9	2.5

n = 15 replicates	Avg	STDdev	%CV
	**199.6**	**6.9**	**3.5**

I.S. LYSINE C13, N15 (MSK-A2)

STD I.S. INTENSITY	REPLICATE A I.S. INTENSITY	REPLICATE B I.S. INTENSITY	REPLICATE C I.S. INTENSITY	SUPPR. A	SUPPR. B	SUPPR. C	*SUPPR. Avg*
1168628.4	889036.4	897312.1	902746	23.9	23.2	22.8	23.3
309194.5	674827.2	763291.5	681467.5	-118.3	-146.9	-120.4	-128.5
922193.4	515363.7	639127.1	723274.1	44.1	30.7	21.6	32.1
1255299.9	775354.2	855328.1	914364.8	38.2	31.9	27.2	32.4
1250600.2	881581	787760	1073929.7	29.5	37.0	14.1	26.9

Samples consist of pooled plasma, urine, or CSF, constituting equal volume mixtures from 5-6 separate patients. Samples were analyzed in triplicate and run in 5 separate batches over the course of approximately 1 week, using fresh aliquots of plasma, urine, and CSF stored at -80°C for each run. The IEX reference value reflects single analysis of an aliquot of pooled sample using ion-exchange chromatography (Biochrom Analyzer). Differences in results between the platforms will depend on matrix-specific features and day-to-day performance, including but not limited to quality of analyte separation during IEX analysis and underlying interferents in the pooled samples affecting optical-based detection. Data for aspartic acid and proline are shown for urine due to relevance of the C13,N15 internal standards of these targets to ornithine (Pro closest in retention time, Asp appeared most similar overall to Orn as internal control during initial testing). In the matrix suppresion analysis, suppression = (STD I.S. Intensity – Replicate I.S. Intensity) / (STD I.S. Intensity). Run 2 was a single outlier for which suppression of internal standard signal in 0.1 M HCl compared to matrix was not observed. For the remaining runs, suppression was generally greater in the pooled plasma sample compared to urine and CSF. Sample preparation error (*) resulted in no chromatography peaks in one CSF sample analysis (Run 2, Replicate C).

Table S13. Matrix-specific precision and signal suppression results (Page 3 of 5).

TESTING IN PLASMA (CONTINUED)

METHIONINE IN PLASMA (IEX REFERENCE VALUE = 99.8 uM)

Run	Replicate A	Replicate B	Replicate C	Avg	STDdev	%CV (Intrarun)
1	97.8	100.7	96.5	98.3	2.2	2.2
2	101.2	102.9	103.1	102.4	1.0	1.0
3	108.3	109.2	105.5	107.7	1.9	1.8
4	103.8	102.6	102.4	102.9	0.8	0.8
5	101.6	108.7	111.2	107.2	4.9	4.6

n = 15 replicates	Avg	STDdev	%CV
	**103.7**	**4.2**	**4.0**

I.S. METHIONINE C13, N15 (MSK-A2)

Run	STD I.S. INTENSITY	REPLICATE A I.S. INTENSITY	REPLICATE B I.S. INTENSITY	REPLICATE C I.S. INTENSITY	SUPPR. A	SUPPR. B	SUPPR. C	*SUPPR. Avg*
1	4404821	3213930.3	3317364.1	3357538.5	27.0	24.7	23.8	25.2
2	1060474.8	2324063.5	2574141.8	2361037	-119.2	-142.7	-122.6	-128.2
3	3311777.4	1799843.6	2156535.9	2566982.8	45.7	34.9	22.5	34.3
4	4825314.6	2998847	3323710.5	3600653.6	37.9	31.1	25.4	31.5
5	5113671.5	3558684.1	3146422.8	4309339.4	30.4	38.5	15.7	28.2

ORNITHINE IN PLASMA (IEX REFERENCE VALUE = 77.7)

Run	Replicate A	Replicate B	Replicate C	Avg	STDdev	%CV (Intrarun)
1	85.35	78.73	74.99	79.7	5.2	6.6
2	80.87	82.79	81.44	81.7	1.0	1.2
3	80.57	79.8	80.7	80.4	0.5	0.6
4	*77.66*	77.66	77.66	77.7	0.0	0.0
5	77.33	79.11	75.83	77.4	1.6	2.1

n = 15 replicates	Avg	STDdev	%CV
	**79.4**	**2.7**	**3.4**

PHENYLALANINE IN PLASMA (IEX REFERENCE VALUE = 104.1 uM)

Run	Replicate A	Replicate B	Replicate C	Avg	STDdev	%CV (Intrarun)
1	105.3	107.0	110.2	107.5	2.5	2.3
2	115.3	115.1	118.7	116.4	2.0	1.7
3	101.7	100.1	101.7	101.2	0.9	0.9
4	119.8	117.6	113.7	117.0	3.1	2.7
5	113.4	116.2	118.9	116.2	2.7	2.4

n = 15 replicates	Avg	STDdev	%CV
	**111.6**	**6.8**	**6.1**

I.S. PHENYLALANINE C13, N15 (MSK-A2)

Run	STD I.S. INTENSITY	REPLICATE A I.S. INTENSITY	REPLICATE B I.S. INTENSITY	REPLICATE C I.S. INTENSITY	SUPPR. A	SUPPR. B	SUPPR. C	*SUPPR. Avg*
1	5868915.6	4692072.5	5035353.4	4823129.6	20.1	14.2	17.8	17.4
2	***1573583***	3304322.9	3716082.7	3525951.1	-110.0	-136.2	-124.1	-123.4
3	4288457.8	2404498.3	3073748.4	3421961.8	43.9	28.3	20.2	30.8
4	6569562.1	4075176.1	4601888.9	4929103.9	38.0	30.0	25.0	31.0
5	7327580.4	4984487.3	4460404.6	5966393.2	32.0	39.1	18.6	29.9

PROLINE IN PLASMA (IEX REFERENCE VALUE = 237.9 uM)

Run	Replicate A	Replicate B	Replicate C	Avg	STDdev	%CV (Intrarun)
1	249.2	235.4	259.9	248.2	12.3	4.9
2	251.5	243.0	246.7	247.1	4.3	1.7
3	247.5	247.8	244.2	246.5	2.0	0.8
4	237.2	248.0	239.6	241.6	5.7	2.4
5	238.5	242.2	226.0	235.6	8.5	3.6

n = 15 replicates	Avg	STDdev	%CV
	**243.8**	**7.9**	**3.3**

I.S. PROLINE C13, N15 (MSK-A2)

Run	STD I.S. INTENSITY	REPLICATE A I.S. INTENSITY	REPLICATE B I.S. INTENSITY	REPLICATE C I.S. INTENSITY	SUPPR. A	SUPPR. B	SUPPR. C	*SUPPR. Avg*
1	2799681.3	1878364.1	2068509	1744202.2	32.9	26.1	37.7	32.2
2	615401.1	1303126.6	1533661.2	1418999.8	-111.8	-149.2	-130.6	-130.5
3	2185076.3	1105969.9	1334793.1	1581359.7	49.4	38.9	27.6	38.6
4	2795335.6	***1628504***	1822940	1928420.2	41.7	34.8	31.0	35.8
5	2966229.5	1819113.7	1691658.1	2386218	38.7	43.0	19.6	33.7

SERINE IN PLASMA (IEX REFERENCE VALUE = 165.2 uM)

Run	Replicate A	Replicate B	Replicate C	Avg	STDdev	%CV (Intrarun)
1	161.9	162.9	158.7	161.2	2.2	1.3
2	175.0	180.0	177.1	177.4	2.5	1.4
3	179.8	182.1	171.4	177.8	5.6	3.2
4	194.9	177.8	186.1	186.2	8.5	4.6
5	191.6	193.4	200.4	195.1	4.6	2.4

n = 15 replicates	Avg	STDdev	%CV
	**179.5**	**12.5**	**6.9**

I.S. SERINE C13, N15 (MSK-A2)

Run	STD I.S. INTENSITY	REPLICATE A I.S. INTENSITY	REPLICATE B I.S. INTENSITY	REPLICATE C I.S. INTENSITY	SUPPR. A	SUPPR. B	SUPPR. C	*SUPPR. Avg*
1	2912785.3	2030774.2	2087198.1	2178665.2	30.3	28.3	25.2	27.9
2	658365.2	1466282.9	1578719.5	1471723.5	-122.7	-139.8	-123.5	-128.7
3	2755233.6	1579649.7	1913079.3	2189425.6	42.7	30.6	20.5	31.3
4	3423052	1832569.6	2137418.9	2261255.2	46.5	37.6	33.9	39.3
5	3373275.8	1996637.6	1801911.8	2518114.1	40.8	46.6	25.4	37.6

THREONINE IN PLASMA (IEX REFERENCE VALUE = 164.7 uM)

Run	Replicate A	Replicate B	Replicate C	Avg	STDdev	%CV (Intrarun)
1	154.6	150.3	146.4	150.4	4.1	2.7
2	156.7	159.5	167.4	161.2	5.5	3.4
3	169.9	167.2	171.5	169.5	2.2	1.3
4	157.1	164.3	170.3	163.9	6.6	4.0
5	169.2	162.7	174.7	168.8	6.0	3.6

n = 15 replicates	Avg	STDdev	%CV
	**162.8**	**8.4**	**5.1**

I.S. THREONINE C13, N15 (MSK-A2)

Run	STD I.S. INTENSITY	REPLICATE A I.S. INTENSITY	REPLICATE B I.S. INTENSITY	REPLICATE C I.S. INTENSITY	SUPPR. A	SUPPR. B	SUPPR. C	*SUPPR. Avg*
1	3165128.1	2642323.2	2747697.6	2690742	16.5	13.2	15.0	14.9
2	760927.6	1748821.4	1902320.3	1735271.3	-129.8	-150.0	-128.0	-136.0
3	2755233.6	1579649.7	1913079.3	2189425.6	42.7	30.6	20.5	31.3
4	3684818.5	2246504.9	2281936.7	2456307.6	39.0	38.1	33.3	36.8
5	3362484.3	2375063.6	2255592.5	3001100.1	29.4	32.9	10.7	24.3

TRYPTOPHAN IN PLASMA (IEX REFERENCE VALUE =74.4)

Run	Replicate A	Replicate B	Replicate C	Avg	STDdev	%CV (Intrarun)
1	98.7	***91.33***	**90.28**	93.4	4.6	4.9
2	**87.78**	87.36	89.81	88.3	1.3	1.5
3	98.25	110.6	105.73	104.9	6.2	5.9
4	**101.14**	101.45	104.35	102.3	1.8	1.7
5	**90.9**	94.76	89.04	91.6	2.9	3.2

n = 15 replicates	Avg	STDdev	%CV
	**96.1**	**7.4**	**7.7**

Samples consist of pooled plasma, urine, or CSF, constituting equal volume mixtures from 5-6 separate patients. Samples were analyzed in triplicate and run in 5 separate batches over the course of approximately 1 week, using fresh aliquots of plasma, urine, and CSF stored at -80°C for each run. The IEX reference value reflects single analysis of an aliquot of pooled sample using ion-exchange chromatography (Biochrom Analyzer). Differences in results between the platforms will depend on matrix-specific features and day-to-day performance, including but not limited to quality of analyte separation during IEX analysis and underlying interferents in the pooled samples affecting optical-based detection. Data for aspartic acid and proline are shown for urine due to relevance of the C13,N15 internal standards of these targets to ornithine (Pro closest in retention time, Asp appeared most similar overall to Orn as internal control during initial testing). In the matrix suppresion analysis, suppression = (STD I.S. Intensity – Replicate I.S. Intensity) / (STD I.S. Intensity). Run 2 was a single outlier for which suppression of internal standard signal in 0.1 M HCl compared to matrix was not observed. For the remaining runs, suppression was generally greater in the pooled plasma sample compared to urine and CSF. Sample preparation error (*) resulted in no chromatography peaks in one CSF sample analysis (Run 2, Replicate C).

Table S13. Matrix-specific precision and signal suppression results (Page 4 of 5).

TESTING IN PLASMA (CONTINUED)

TYROSINE IN PLASMA (IEX REFERENCE VALUE = 105.0 uM)

Run	Replicate A	Replicate B	Replicate C	Avg	STDdev	%CV (Intrarun)
1	110.4	112.7	119.8	114.3	4.9	4.3
2	117.2	115.3	123.0	118.5	4.0	3.4
3	110.9	114.1	111.4	112.1	1.7	1.5
4	112.8	120.1	111.2	114.7	4.8	4.2
5	110.7	114.1	118.2	114.3	3.8	3.3

n = 15 replicates	Avg	STDdev	%CV
	114.8	4.0	3.5

I.S. TYROSINE C13, N15 (MSK-A2)

STD I.S. INTENSITY	REPLICATE A I.S. INTENSITY	REPLICATE B I.S. INTENSITY	REPLICATE C I.S. INTENSITY	SUPPR. A	SUPPR. B	SUPPR. C	*SUPPR. Avg*
3185544.4	2490174.2	2717887.6	2543020.7	21.8	14.7	20.2	18.9
870659.5	1890728.9	2063104.1	1887238.2	-117.2	-137.0	-116.8	-123.6
2529882.7	1397339.3	1738543.9	2012331.3	44.8	31.3	20.5	32.2
3669464.8	2399801.7	2660933.8	2955443.2	34.6	27.5	19.5	27.2
4184592.1	2938263.7	2436975.1	3418322.5	29.8	41.8	18.3	30.0

VALINE IN PLAMSA (IEX REFERENCE VALUE = 313.4 uM)

Run	Replicate A	Replicate B	Replicate C	Avg	STDdev	%CV (Intrarun)
1	298.3	318.2	320.9	312.5	12.4	4.0
2	324.2	320.4	316.1	320.2	4.1	1.3
3	315.0	334.1	333.1	327.4	10.7	3.3
4	318.0	308.2	323.5	316.6	7.8	2.5
5	330.0	322.6	319.6	324.1	5.3	1.6

n = 15 replicates	Avg	STDdev	%CV
	320.1	9.1	2.8

I.S. VALINE C13, N15 (MSK-A2)

STD I.S. INTENSITY	REPLICATE A I.S. INTENSITY	REPLICATE B I.S. INTENSITY	REPLICATE C I.S. INTENSITY	SUPPR. A	SUPPR. B	SUPPR. C	*SUPPR. Avg*
3205994.9	2456237.8	2493833.2	2358422.1	23.4	22.2	26.4	24.0
743450	1637249.9	1781507.7	1718047.1	-120.2	-139.6	-131.1	-130.3
2130742.7	1147819.6	1456864.3	1690435.6	46.1	31.6	20.7	32.8
3794175.6	2359681.4	2753936.6	2817267.2	37.8	27.4	25.7	30.3
3720656.4	2679521.8	2334126.8	3343633.7	28.0	37.3	10.1	25.1

TESTING IN URINE (TARGET ANALYTES = ARGININE, LYSINE AND ORNITHINE)

ARGININE IN URINE (IEX REFERENCE VALUE = 8.2 uM)

Run	Replicate A	Replicate B	Replicate C	Avg	STDdev	%CV (Intrarun)
1	31.89	30.02	30.19	30.7	1.0	3.4
2	29.7	31.45	29.06	30.1	1.2	4.1
3	32.07	33.43	35.21	33.6	1.6	4.7
4	36.62	35.4	34.34	35.5	1.1	3.2
5	33.92	32.67	33.38	33.3	0.6	1.9

n = 15 replicates	Avg	STDdev	%CV
	32.6	2.3	7.0

I.S. ARGININE C13, N15 (MSK-A2)

STD I.S. INTENSITY	REPLICATE A I.S. INTENSITY	REPLICATE B I.S. INTENSITY	REPLICATE C I.S. INTENSITY	SUPPR. A	SUPPR. B	SUPPR. C	*SUPPR. Avg*
2548978.6	2466282.6	2606391.9	2605059.6	3.2	-2.3	-2.2	-0.4
664968.5	1876040.3	1872587.8	1804798.7	-182.1	-181.6	-171.4	-178.4
1834163.4	1781742	1692134.8	1874465.1	2.9	7.7	-2.2	2.8
2476249.2	1995746.4	2175603.4	2021436	19.4	12.1	18.4	16.6
2344328.8	3111065.5	2766805.5	2747534.5	-32.7	-18.0	-17.2	-22.6

ASPARTIC ACID IN URINE (IEX REFERENCE VALUE = 8.87 uM)

Run	Replicate A	Replicate B	Replicate C	Avg	STDdev	%CV (Intrarun)
1	8.35	8.27	7.79	8.1	0.3	3.7
2	7.04	8.19	8.28	7.8	0.7	8.8
3	7.9	7.75	8.06	7.9	0.2	2.0
4	9.21	9.72	8.53	9.2	0.6	6.5
5	8.62	7.86	7.93	8.1	0.4	5.2

n = 15 replicates	Avg	STDdev	%CV
	8.2	0.6	7.7

I.S. ASPARTIC ACID C13, N15 (MSK-A2)

STD I.S. INTENSITY	REPLICATE A I.S. INTENSITY	REPLICATE B I.S. INTENSITY	REPLICATE C I.S. INTENSITY	SUPPR. A	SUPPR. B	SUPPR. C	*SUPPR. Avg*
2336347	2623649.4	2397922.9	2535981	-12.3	-2.6	-8.5	-7.8
553561	1612672.8	1566381.6	1462755.5	-191.3	-183.0	-164.2	-179.5
2581749.8	2272603.7	2260103.8	2433604.5	12.0	12.5	5.7	10.1
2631483	2143552.4	2187881.5	2117917.4	18.5	16.9	19.5	18.3
2618287.5	2586703.6	2293713.4	2446328	1.2	12.4	6.6	6.7

LYSINE IN URINE (IEX REFERENCE VALUE = 168.55 uM)

Run	Replicate A	Replicate B	Replicate C	Avg	STDdev	%CV (Intrarun)
1	133.46	130.2	132.63	132.1	1.7	1.3
2	128.04	125.12	122.68	125.3	2.7	2.1
3	132.05	127.37	125.67	128.4	3.3	2.6
4	141.74	139.55	145	142.1	2.7	1.9
5	130.27	135.02	134.46	133.3	2.6	1.9

n = 15 replicates	Avg	STDdev	%CV
	132.2	6.3	4.8

I.S. LYSINE C13, N15 (MSK-A2)

STD I.S. INTENSITY	REPLICATE A I.S. INTENSITY	REPLICATE B I.S. INTENSITY	REPLICATE C I.S. INTENSITY	SUPPR. A	SUPPR. B	SUPPR. C	*SUPPR. Avg*
1168628.4	1222650.6	1136282.2	1206968.1	-4.6	2.8	-3.3	-1.7
309194.5	886775.7	858622.5	807570.1	-186.8	-177.7	-161.2	-175.2
922193.4	787390.4	768191.7	857424.1	14.6	16.7	7.0	12.8
1255299.9	1057009.4	1080410.4	1025004.5	15.8	13.9	18.3	16.0
1250600.2	1248210.3	1057268.5	1079881.6	0.2	15.5	13.7	9.8

ORNITHINE IN URINE (IEX REFERENCE VALUE = 9.96)

Run	Replicate A	Replicate B	Replicate C	Avg	STDdev	%CV (Intrarun)
1	11.8	12.23	11.09	11.7	0.6	4.9
2	9.71	10.46	10.2	10.1	0.4	3.8
3	10	10.31	***10.94***	10.4	0.5	4.6
4	13.01	14.92	12.52	13.5	1.3	9.4
5	12.68	11.33	11.46	11.8	0.7	6.3

n = 15 replicates	Avg	STDdev	%CV
	11.5	1.4	12.1

PROLINE IN URINE (IEX REFERENCE VALUE = 0 uM)

Run	Replicate A	Replicate B	Replicate C	Avg	STDdev	%CV (Intrarun)
1	7.61	7.41	8.02	7.7	0.3	4.0
2	7.13	7.33	7.62	7.4	0.2	3.3
3	7.28	7.65	8.01	7.6	0.4	4.8
4	7.94	8.13	7.39	7.8	0.4	4.9
5	7.59	6.94	6.71	7.1	0.5	6.4

n = 15 replicates	Avg	STDdev	%CV
	7.5	0.4	5.4

I.S. PROLINE C13, N15 (MSK-A2)

STD I.S. INTENSITY	REPLICATE A I.S. INTENSITY	REPLICATE B I.S. INTENSITY	REPLICATE C I.S. INTENSITY	SUPPR. A	SUPPR. B	SUPPR. C	*SUPPR. Avg*
2799681.3	2425942.7	2472741.4	2538339.7	13.3	11.7	9.3	11.5
615401.1	***1806015.3***	1720068.2	1568481.3	-193.5	-179.5	-154.9	-175.9
2185076.3	1742163.8	1682722.1	1960518.9	20.3	23.0	10.3	17.8
2795335.6	***2269531***	2259389	2204010.7	18.8	19.2	21.2	19.7
2966229.5	2711365.1	2604399.7	2708621.4	8.6	12.2	8.7	9.8

Samples consist of pooled plasma, urine, or CSF, constituting equal volume mixtures from 5-6 separate patients. Samples were analyzed in triplicate and run in 5 separate batches over the course of approximately 1 week, using fresh aliquots of plasma, urine, and CSF stored at -80°C for each run. The IEX reference value reflects single analysis of an aliquot of pooled sample using ion-exchange chromatography (Biochrom Analyzer). Differences in results between the platforms will depend on matrix-specific features and day-to-day performance, including but not limited to quality of analyte separation during IEX analysis and underlying interferents in the pooled samples affecting optical-based detection. Data for aspartic acid and proline are shown for urine due to relevance of the C13,N15 internal standards of these targets to ornithine (Pro closest in retention time, Asp appeared most similar overall to Orn as internal control during initial testing). In the matrix suppresion analysis, suppression = (STD I.S. Intensity – Replicate I.S. Intensity) / (STD I.S. Intensity). Run 2 was a single outlier for which suppression of internal standard signal in 0.1 M HCl compared to matrix was not observed. For the remaining runs, suppression was generally greater in the pooled plasma sample compared to urine and CSF. Sample preparation error (*) resulted in no chromatography peaks in one CSF sample analysis (Run 2, Replicate C).

Table S13. Matrix-specific precision and signal suppression results (Page 5 of 5).

TESTING IN CSF (TARGET ANALYTES = GLYCINE AND SERINE)

GLYCINE IN CSF (IEX REFERENCE VALUE = 62.2 uM)

Run	Replicate A	Replicate B	Replicate C	Avg	STDdev	%CV (Intrarun)
1	66.0	65.0	63.6	64.8	1.2	1.9
2	70.7	67.5	*	69.1	2.3	3.3
3	58.4	60.6	60.8	59.9	1.3	2.3
4	70.5	67.4	66.4	68.1	2.1	3.1
5	66.9	59.7	57.7	61.4	4.8	7.8

I.S. GLYCINE C13, N15 (MSK-A2)

Run	STD I.S. INTENSITY	REPLICATE A I.S. INTENSITY	REPLICATE B I.S. INTENSITY	REPLICATE C I.S. INTENSITY	SUPPR. A	SUPPR. B	SUPPR. C	*SUPPR. Avg*
1	3637497.2	3652634.4	3426375	3555922.9	-0.4	5.8	2.2	2.5
2	820876.3	1993137.2	2409282.6	*	-142.8	-193.5	*	-168.2
3	4370385.6	3949037.9	3964922	4109487.1	9.6	9.3	6.0	8.3
4	4060489.5	3114442.7	3588391.2	*3546863.4*	23.3	11.6	12.6	15.9
5	4053265.6	4423952.1	4934997.4	5566313.4	-9.1	-21.8	-37.3	-22.7

n = 14 replicates	Avg	STDdev	%CV
	**64.4**	**4.3**	**6.7**

SERINE IN CSF (IEX REFERENCE VALUE = 34.3 uM)

Run	Replicate A	Replicate B	Replicate C	Avg	STDdev	%CV (Intrarun)
1	32.6	32.1	36.1	33.6	2.2	6.6
2	37.3	38.0	*	37.6	0.5	1.3
3	35.6	37.5	38.8	37.3	1.6	4.3
4	35.3	37.8	40.8	38.0	2.7	7.2
5	43.5	42.7	39.6	42.0	2.1	4.9

I.S. SERINE C13, N15 (MSK-A2)

Run	STD I.S. INTENSITY	REPLICATE A I.S. INTENSITY	REPLICATE B I.S. INTENSITY	REPLICATE C I.S. INTENSITY	SUPPR. A	SUPPR. B	SUPPR. C	*SUPPR. Avg*
1	2912785.3	2968357.2	2919708.2	2749405.9	-1.9	-0.2	5.6	1.2
2	658365.2	1550758.9	1731617.8	*	-135.5	-163.0	*	-149.3
3	2755233.6	2583947.7	2666857.9	2747504.8	6.2	3.2	0.3	3.2
4	3423052	2464538.4	2703154.4	2678801.5	28.0	21.0	21.7	23.6
5	3373275.8	2721142.5	2736578.3	2919810	19.3	18.9	13.4	17.2

n = 14 replicates	Avg	STDdev	%CV
	**37.7**	**3.3**	**8.8**

Samples consist of pooled plasma, urine, or CSF, constituting equal volume mixtures from 5-6 separate patients. Samples were analyzed in triplicate and run in 5 separate batches over the course of approximately 1 week, using fresh aliquots of plasma, urine, and CSF stored at -80°C for each run. The IEX reference value reflects single analysis of an aliquot of pooled sample using ion-exchange chromatography (Biochrom Analyzer). Differences in results between the platforms will depend on matrix-specific features and day-to-day performance, including but not limited to quality of analyte separation during IEX analysis and underlying interferents in the pooled samples affecting optical-based detection. Data for aspartic acid and proline are shown for urine due to relevance of the C13,N15 internal standards of these targets to ornithine (Pro closest in retention time, Asp appeared most similar overall to Orn as internal control during initial testing). In the matrix suppresion analysis, suppression = (STD I.S. Intensity – Replicate I.S. Intensity) / (STD I.S. Intensity). Run 2 was a single outlier for which suppression of internal standard signal in 0.1 M HCl compared to matrix was not observed. For the remaining runs, suppression was generally greater in the pooled plasma sample compared to urine and CSF. Sample preparation error (*) resulted in no chromatography peaks in one CSF sample analysis (Run 2, Replicate C).

Table S14. Lipemia mixing studies (plasma).

AA	Unmixed LC-MS/MS (µM)						Mixed A+F			Mixed B+F			Mixed C+F			Mixed D+F			Mixed E+F		
	A	B	C	D	E	F	Result (µM)	Exp. (µM)	Diff. (%)	Result (µM)	Exp. (µM)	Diff. (%)	Result (µM)	Exp. (µM)	Diff. (%)	Result (µM)	Exp. (µM)	Diff. (%)	Result (µM)	Exp. (µM)	Diff. (%)
Tau	53	16	104	21	137	126	87	90	-3.4	78	71	9.0	100	115	-15.0	70	74	-5.7	156	132	15.4
PEA	<5	<5	<5	<5	<5	<5	<5	<5	N/A	<5	<5	N/A	<5	<5	N/A	<5	<5	N/A	<5	<5	N/A
Asp	6	7	14	<5	14	16	12	11	8.3	12	12	0.0	15	15	0.0	10	11	-10.0	15	15	0.0
Thr	95	56	161	286	209	172	134	134	0.0	113	114	-0.9	169	167	1.2	251	229	8.8	195	191	2.1
Ser	137	83	95	133	92	168	160	153	4.4	134	126	6.0	128	132	-3.1	157	151	3.8	133	130	2.3
Asn	81	51	57	<5	59	64	79	73	7.6	54	58	-7.4	62	61	1.6	33	35	-6.1	66	62	6.1
Glu	90	145	252	441	240	46	66	68	-3.0	85	96	-12.9	144	149	-3.5	237	244	-3.0	141	143	-1.4
Gln	609	369	402	<5	391	504	534	554	-3.7	391	437	-11.8	423	453	-7.1	230	255	-10.9	404	448	-10.9
Gly	159	273	168	266	158	498	318	329	-3.5	338	386	-14.2	328	333	-1.5	348	382	-9.8	322	328	-1.9
Ala	300	377	274	640	331	283	283	292	-3.2	315	330	-4.8	282	279	1.1	451	462	-2.4	311	307	1.3
Cit	15	10	12	11	15	27	20	21	-5.0	16	19	-18.8	18	20	-11.1	19	19	0.0	20	21	-5.0
Val	167	166	212	333	276	140	154	154	0.0	152	153	-0.7	178	176	1.1	239	237	0.8	209	208	0.5
Cys	11	11	67	16	68	10	10	11	-10.0	9	11	-22.2	37	39	-5.4	11	13	-18.2	45	39	13.3
Met	22	9	20	21	28	26	23	24	-4.3	17	18	-5.9	24	23	4.2	23	24	-4.3	28	27	3.6
aIle	<5	<5	<5	<5	<5	<5	<5	<5	N/A	<5	<5	N/A	<5	<5	N/A	<5	<5	N/A	<5	<5	N/A
Ile	65	23	98	76	132	49	57	57	0.0	34	36	-5.9	77	74	3.9	66	63	4.5	96	91	5.2
Leu	103	62	134	178	183	95	100	99	1.0	73	79	-8.2	115	115	0.0	141	137	2.8	148	139	6.1
ASA	<5	<5	<5	<5	<5	<5	<5	<5	N/A	<5	<5	N/A	<5	<5	N/A	<5	<5	N/A	<5	<5	N/A
Tyr	63	43	53	47	74	34	43	49	-14.0	36	39	-8.3	44	44	0.0	39	41	-5.1	55	54	1.8
Phe	36	31	59	186	67	482	273	259	5.1	245	257	-4.9	308	271	12.0	336	334	0.6	295	275	6.8
HCY	<5	<5	<5	<5	<5	<5	<5	<5	N/A	<5	<5	N/A	<5	<5	N/A	<5	<5	N/A	<5	<5	N/A
Orn	89	52	94	71	100	73	77	81	-5.2	61	63	-3.3	78	84	-7.7	76	72	5.3	85	87	-2.4
Lys	137	91	170	126	204	101	126	119	5.6	91	96	-5.5	144	134	6.9	109	114	-4.6	165	153	7.3
His	81	65	65	98	72	75	76	78	-2.6	66	70	-6.1	69	70	-1.4	86	87	-1.2	73	74	-1.4
Trp	59	22	49	19	50	33	46	46	0.0	27	28	-3.7	41	41	0.0	26	26	0.0	42	42	0.0
Arg	111	5	65	36	60	74	89	93	-4.5	37	40	-8.1	64	70	-9.4	50	55	-10.0	63	67	-6.3
Pro	187	178	274	241	394	119	152	153	-0.7	143	149	-4.2	192	197	-2.6	193	180	6.7	274	257	6.2
HCT	<5	<5	<5	<5	<5	<5	<5	<5	N/A	<5	<5	N/A	<5	<5	N/A	<5	<5	N/A	<5	<5	N/A

Sample A Trig = 445 mg/dL, Sample B Trig = 1151 mg/dL, Sample C Trig = 1081 mg/dL. Sample D Trig = 1588 mg/dL, Sample E Trig = 1756 mg/dL, Sample F Trig = 52 mg/dL
